# Global prevalence of fatigue in patients with multiple sclerosis: a systematic review and meta-analysis

**DOI:** 10.3389/fneur.2024.1457788

**Published:** 2024-10-02

**Authors:** Xiaodong Yi, Yue Zhang, Qiufeng Du, Jing Kang, Shuang Song, Tao Li, Yunlan Jiang

**Affiliations:** ^1^College of Nursing, Chengdu University of Traditional Chinese Medicine, Chengdu, China; ^2^Hospital of Chengdu University of Traditional Chinese Medicine, Chengdu, Sichuan, China

**Keywords:** fatigue, prevalence, multiple sclerosis, systematic review, meta-analysis

## Abstract

**Background:**

Fatigue is one of the most common and burdensome symptoms for patients with multiple sclerosis (PwMS), considerably impacting their quality of life and employment. Numerous reports have described the prevalence of MS-related fatigue, but there is no global consensus on this matter.

**Objective:**

To examine the global prevalence of MS-related fatigue and identify sources of heterogeneity in the published literature.

**Methods:**

A systematic review and meta-analysis were conducted. A comprehensive search of the PubMed, EMBASE, Cochrane Library, Web of Science, PsycINFO, CINAHL, China National Knowledge Infrastructure (CNKI), and Wanfang database for potential literature from 2000 to January 31, 2024. A random effects model was used to calculate the prevalence of MS-related fatigue. Subgroup analyses and a meta-regression were used to explore the sources of heterogeneity.

**Results:**

Sixty-nine studies from 27 countries were included. The global prevalence of MS-related fatigue was 59.1%, and it has decreased every decade since 2000. Fatigue was prevalent among females, those with lower education levels, those who were older, those with greater disability, and those with longer MS durations. The meta-regression revealed that fatigue measurement instruments were the largest source of heterogeneity.

**Conclusion:**

The prevalence of MS-related fatigue is quite high. Healthcare professionals should screen for and manage fatigue for PwMS as early as possible and pay attention to populations with a high prevalence of fatigue. The high heterogeneity among the prevalence rates due to differences in the fatigue scales suggests the importance of reaching a consensus on the best screening tools for MS-related fatigue.

## 1 Introduction

Multiple sclerosis (MS) is a chronic, inflammatory, and demyelinating disease that affects the central nervous system and is one of the most common causes of nontraumatic disability among young adults (1, 2). The reported prevalence of MS increased in every WHO region between the 2013 and 2020 versions of the Atlas of MS (3). A total of 2.8 million people worldwide have MS, with the highest rates reported in the WHO European Region (EUR) and Region of the Americas (AMR) and the lowest rates reported in the WHO African Region (AFR) and Western Pacific Region (WPR) (2). MS is divided into three phenotypes according to the disease activity, progression, and clinical course: relapsing–remitting MS (RRMS), secondary progressive MS (SPMS), and primary progressive MS (PPMS) (4). Approximately 85% of PwMS are initially diagnosed with RRMS and develop SPMS with or without superimposed relapses over time (5, 6). PwMS display a diverse range of signs and symptoms, varying in severity, stemming from damage to the central nervous system. These include but are not limited to spasticity, pain, fatigue, bladder and bowel issues, gait impairments, mood disturbances, and sleep disorders (7).

Fatigue is one of the most common and burdensome symptoms of MS (8), manifesting at any given stage and timepoint throughout the course of the disease (9). It is conceptualized as “a significant lack of physical and/or mental energy, perceived by the individual or caregiver, that interferes with normal and desired activities” (9). The exact pathophysiology of MS-related fatigue remains unclear (10), but it is usually classified into primary and secondary fatigue to better understand this symptom. Primary fatigue, which is considered to be specific to MS, refers to fatigue that occurs without an obvious cause and is a direct consequence of the primary pathological mechanisms of MS (11, 12). In general, secondary fatigue is caused by sleep disturbances, mood disorders (anxiety and depression), side effects of disease-modifying treatments (DMT) and decreased physical activity (10). MS-related fatigue is a complex phenomenon (13) that differs from the fatigue observed in healthy individuals because of its disabling nature and inability to be relieved by rest or sleep (14). Patients with MS-related fatigue execute daily tasks with an unduly high level of effort, as reflected by a reduced ability to perform daily living and work. In PwMS, fatigue is one of the main drivers of low health-related QoL and unemployment (10, 15, 16). Fatigue is also a major predictor of claims for social benefits such as sick leave and disability pensions (13). Hence, fatigue represents one of the most urgent clinical problems in the treatment and management of MS (17).

Currently, there are no convincing pharmacological treatments available for MS-related fatigue (9, 10). Despite this, over the years, due to the continued efforts of MS practitioners, patients with fatigue can benefit from nonpharmacological interventions, including physical activity, dietary modification and cognitive behavioral therapy (10). The prevalence of fatigue may be changing (18) due to the availability of nonpharmacological treatments and advances in the diagnosis and treatment of MS (19). In MS, more than a dozen fatigue questionnaires exist, among which the Fatigue Severity Scale (FSS), Modified Fatigue Impact Scale (MFIS) and Fatigue Scale for Motor and Cognitive Functions (FSMC) are commonly used tools and have been recommended as patient-reported outcomes in clinical trials; these tools measure different dimensions of fatigue and have multiple cut-off values for use (9, 20, 21). Numerous studies describing the prevalence rates of MS-related fatigue have been conducted worldwide. Nevertheless, the results of the studies vary widely, with the prevalence of fatigue ranging from 28.4 to 88.2% (18, 22, 23), probably due to differences in the measurement tools (fatigue scales and cut-off values) and patient characteristics. Up-to-date and accurate prevalence estimates are essential for a clearer understanding of the fatigue burden of MS. A previous systematic literature review (SLR) reported prevalence rates spanning from 36.5 to 78% across 12 studies (6), all of which utilized validated fatigue scales and explicitly reported reference cut-off values. There is still no global consensus on the prevalence of MS-related fatigue, although research on this topic is ongoing. We conducted this systematic review and meta-analysis to determine the global prevalence of MS-related fatigue and identify sources of heterogeneity, with the aim of providing useful indicators and recommendations for policies, programs, relevant agencies and healthcare professionals.

## 2 Methods

### 2.1 Protocol and registration

The study protocol was registered within the PROSPERO international prospective register of systematic reviews (CRD42024499139), and we reported this study following the Preferred Reporting Items for Systematic Reviews and Meta-Analyses (PRISMA) guidelines (24) (Supplementary Table 1).

### 2.2 Search strategy and data sources

A systematic computerized search in the PubMed, EMBASE, Cochrane Library, Web of Science, PsycINFO, CINAHL, CNKI, and Wanfang database was completed on January 31, 2024. Chinese and English language studies on MS-related fatigue published since 2000 were screened. The search strategy was developed in consultation with an expert research librarian and was adjusted accordingly for each database. The medical subject heading (MeSH) terms ‘Multiple Sclerosis,’ ‘Multiple Sclerosis, Relapsing Remitting,’ and ‘Multiple Sclerosis, Chronic Progressive’ were combined with ‘Fatigue’ and with ‘Epidemiology,’ ‘Epidemiologic Studies,’ and ‘Prevalence.’ The search was supplemented with a free text word search of these terms (the electronic search strategy is displayed in Supplementary Table 2).

### 2.3 Eligibility criteria

Publications were rigorously screened based on the following inclusion criteria: (1) observational original studies, encompassing cross-sectional, cohort and case–control studies; (2) subjects aged 17 years and older with an MS diagnosis, either by self-report or by a clinician according to Poser criteria or McDonald criteria (25), either by self-report or by a clinician; (3) full-text publications in a peer-reviewed journal; and (4) studies using a validated fatigue measurement scale with a cut-off value indicating clinically significant fatigue.

The exclusion criteria were as follows: (1) studies that included only patients with clinical isolated syndrome or inpatients; (2) studies in which fatigue was an inclusion criterion; (3) studies in which the sample size was smaller than 100; (4) studies lacking sufficient information for prevalence calculations, such as the total sample and number or percentage of fatigued patients; and (5) studies containing duplicated data.

### 2.4 Study selection

Study screening was performed with Endnote X9 software. Following the removal of duplicate records, two investigators (X.D.Y., Q.F.D.) independently screened all the remaining titles and abstracts on the basis of the selection criteria. The retrieval of the full-text articles was conducted whenever at least one reviewer deemed an abstract suitable for inclusion. Both investigators independently evaluated each publication for final inclusion in the study. Disputes were settled through consensus. In the absence of consensus, a designated author (Y.L.J.) was tasked with making the ultimate decision.

### 2.5 Data extraction

Upon determining the definitive article list, a data-extraction sheet was formulated and underwent a pilot test utilizing 15 pseudorandomly selected articles. Two reviewers (S.S. and Q.F.D. or J.K. and T.L.) used the datasheets (Microsoft Excel) to independently extract the following data from each article: (1) study: first author, year of publication, study design, sample size, patient source, survey year, country, and MS diagnosis criteria; (2) PwMS: age (years), number of males and females, MS duration, MS phenotype, Expanded Disability Status Scale (EDSS) score and level of education; and (3) study outcomes: fatigue cases or prevalence, fatigue scale and cut-off value. For longitudinal studies, we accounted for baseline data. If the survey spanned several years, the average of the survey years was taken.

### 2.6 Risk of bias assessment

Two reviewers (S.S. and Q.F.D. or J.K. and T.L.) independently assessed the risk of bias (ROB) of the included studies, and any discrepancies were resolved through consensus or consultation with a third researcher (Y.L.J.). The methodological quality assessment criteria recommended by the Agency for Healthcare Research and Quality (AHRQ) were employed to evaluate the cross-sectional studies (26). The AHRQ included a total of 11 items, each of which were evaluated as “yes,” “no” or “unclear” and were scored as 1 for “yes” or 0 for “no” or “unclear” (notably, for the fifth item, the scoring was reversed). A cumulative score of 0–3 indicates “low quality,” 4–7 indicates “moderate quality,” and 8–11 indicates “high quality.” The Newcastle–Ottawa Scale (NOS) was used to assess the ROB in the case–control and cohort studies (27). The 8-item scale is constructed to evaluate three major features of the study: sample selection, comparability between groups, and exposure, with a maximum score of 9. The ROB was deemed low for studies with 7–9 stars, moderate for those with 5–6 stars, and high for those with fewer than 4 stars.

### 2.7 Statistical analysis

We executed our data analysis using the R programming language (version 4.2.3) and the “meta” package of RStudio software for Windows (28). As heterogeneity is inherent among prevalence studies, all meta-analyses of MS-related fatigue pooled prevalences and 95% confidence intervals (95% CI) were calculated using a random-effects model. We used the Freeman–Tukey transformation to calculate the weighted pooled prevalence of MS-related fatigue to correct for raw proportions that were nonnormally distributed. The *χ*^2^-based Cochrane Q statistic and *I*^2^ test were used to assess the heterogeneity between studies using two-sided *p* values, with *I*^2^ ≥ 75% indicating considerable heterogeneity. We used subgroup and meta-regression analyses to define the potential sources of heterogeneity. According to study-level characteristics, subgroup analyses were conducted separately by sample size (<1000, >1000), survey year (2000–2009, 2010–2019, 2020–2023), patient source (Academic MS Medical Research Centre, population-based, Outpatient, mixed source patients), age, proportion of females (<60%, 60 ~ 80%, ≥80%), MS duration (≤10 years, >10 years), EDSS score (≤4, >4), fatigue scale, WHO region (AMR, EMR [Eastern Mediterranean Region], EUR, WPR) (29), and income level [upper-middle-income, high-income, based on the gross national income *per capita* level, according to the World Bank criteria published annually (30)]. At least three studies needed to be available per subgroup (31). To determine the degree to which distinct variations in patient or study characteristics influenced the various prevalence estimates, meta-regression analyses were implemented. Univariate meta-regression analyses were conducted on the sample size, survey year, patient source, age, proportion of females, MS duration, EDSS score, fatigue scale, WHO region and income level in the subgroups. Two variables, the EDSS score and MS duration, had 25 and 43 available data points, respectively, in the meta-regression analyses. For the remaining variables, studies were excluded from consideration for the meta-regression analyses if data were missing for a particular variable, and 56 data points were ultimately excluded. For the age variable, missing mean age values were imputed with medians, as previously validated (32). As studies have shown that traditional methods, such as funnel plots and asymmetry tests, are not suitable for assessing publication bias in prevalence studies (33), this study did not evaluate publication bias. In addition, the robustness and stability of the results were assessed by means of a sensitivity analysis that removed studies one by one. For all the tests, we considered *p* < 0.05 to indicate statistical significance.

## 3 Results

### 3.1 Study identification

**Figure 1:**
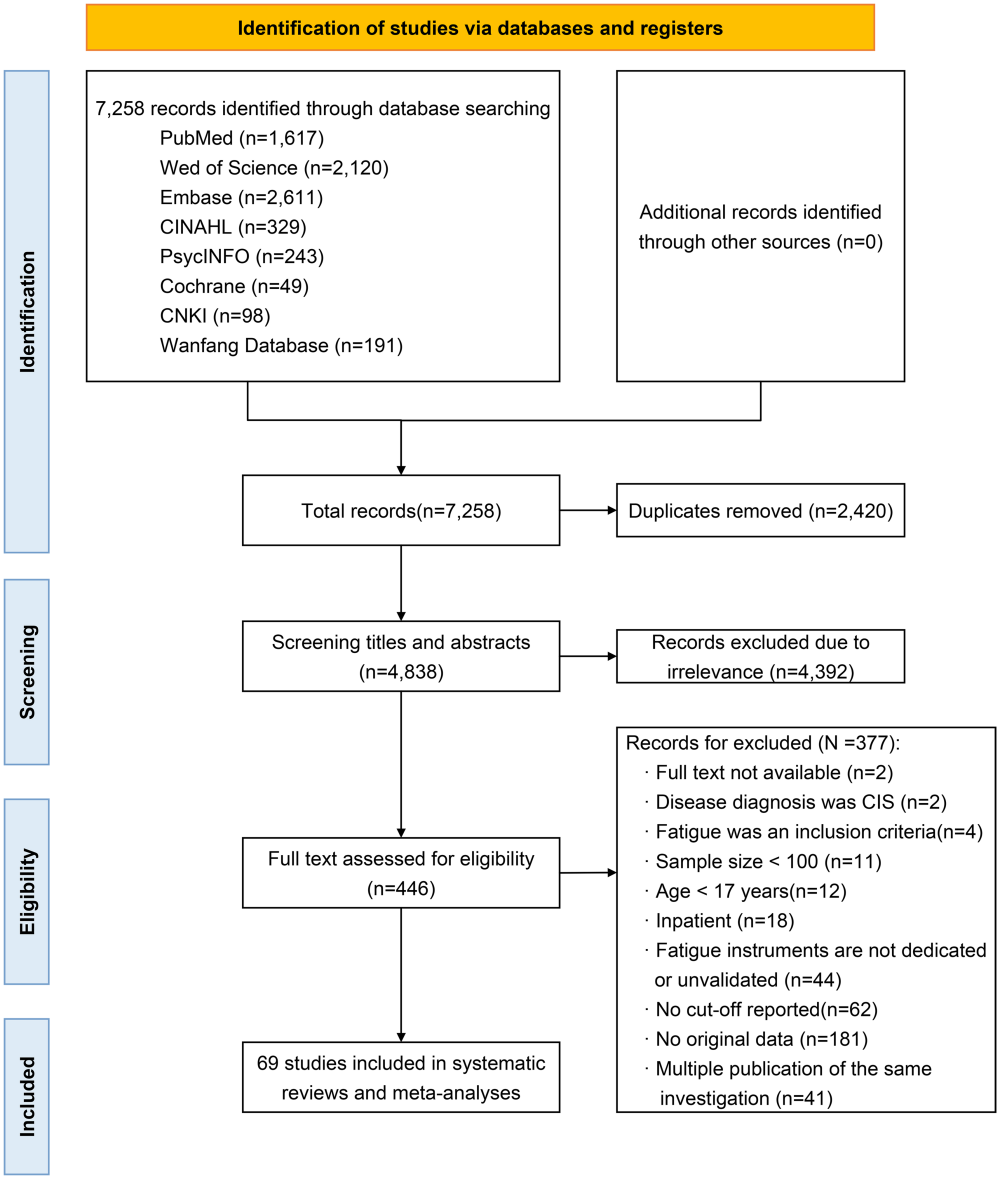
PRISMA diagram of the literature search and study selection.

As depicted in Figure 1, our cross-database search yielded a total of 7,258 records. After eliminating duplicates, 4,838 titles and abstracts were assessed for relevance, with 446 full-text articles screened against the eligibility criteria. Ultimately, 69 studies were included in the final analysis. The complete citation information for the included studies is shown in Supplementary Table 3.

### 3.2 Study and sample characteristics

Sixty-nine studies involving 44,468 PwMS were included in this meta-analysis. The average number of participants per study was 644, varying from 100 to 9,077, the mean age ranged from 32.4 to 59.3 years, the mean EDSS score ranged from 1.9 to 6.5, the mean MS duration ranged from 4.1 to 22.2 years, and the proportion of females ranged from 55 to 86%. All studies spanned the last 21 years (from 2002 onwards) and were conducted in 27 different countries across the four WHO regions. Over 50% of the studies (*n* = 36) were carried out in the EUR, with none from the AFR or the Southeast Asia Region (SEAR). The countries with the greatest number of included studies were the USA (*n* = 9), Argentina (*n* = 5), the UK (*n* = 4), Italy (*n* = 4), Australia (*n* = 4), Saudi Arabia (*n* = 3), the Netherlands (*n* = 3), China (*n* = 3), and Finland (*n* = 3). Two studies were binational, one in the USA and Sweden and the other in Turkey and Israel. In addition, three studies were multinational. According to the World Bank classification, the vast majority of the studies were performed in high-income countries (*n* = 53 studies, 77%), followed by upper-middle-income countries. In terms of patient recruitment, almost half of the studies were population-based (*n* = 34), 24 were performed in MS outpatient clinics, and 4 were performed in MS Medical Research Centres. Most studies had a cross-sectional design (*n* = 64, 93%), 4 had a case–control design, and one had a cohort design. Among the subset of studies examining the prevalence of patient characteristics, 15 studies provided data on sex distribution, 15 studies reported the prevalence of fatigue with different MS phenotypes, and 4 studies estimated the prevalence by education level. Furthermore, 34 studies reported diagnostic criteria for MS, of which two published earlier studies used the previous Poser criteria, and all later studies used the continuously updated McDonald criteria (2001, 2005, 2010, 2017) (25). Further sample and study characteristics can be found in Supplementary Table 4.

### 3.3 Assessment of fatigue in MS

The included studies employed four fatigue scales: two-thirds of the studies used the FSS (*n* = 47), one-fifth the MFIS (*n* = 16), 4 used the FSMC with a cut-off value of 43 (total score), and one used the EMIF-SEP [a validated French version of the Fatigue Impact Scale (34)] with a cut-off value of 55 (total score). Furthermore, a single study used both the FFS and MFIS with cut-off values of 4 (mean score) and 38 (total score), respectively. Of the 47 studies using the FSS, 28 reported fatigue prevalence using a cut-off value of 4 (mean score), 17 used a cut-off value of 5 (mean score), and the remaining two used cut-off values of 4.5 (mean score) and 28 (total score). Of the 16 articles that used the MFIS, 14 used a total score of 38 as the cut-off value, and 2 used total scores of 35.5 and 45 as the cut-off values.

### 3.4 Risk of bias assessment

In all four case–control studies and the one cohort study, the NOS score ranged from 4 to 6 stars, indicating moderate ROB. Using the AHRQ scale, we observed a low ROB among the 64 cross-sectional studies since the mean score was 7.6 (SD = 7.6, range: 5–10). Of the cross-sectional studies, 59% of the included studies were appraised as being of “moderate quality,” and 28 studies (41%) were appraised as “high quality.” The major constraints representing ROB included inadequate details on the quality-control methods for the outcome measures, the use of nonblinded evaluators, and unclear methods for handling missing data in the statistical analyses. The detailed ROB assessments are presented in Supplementary Tables 5, 6.

### 3.5 Overall features of fatigue prevalence

**Figure 2:**
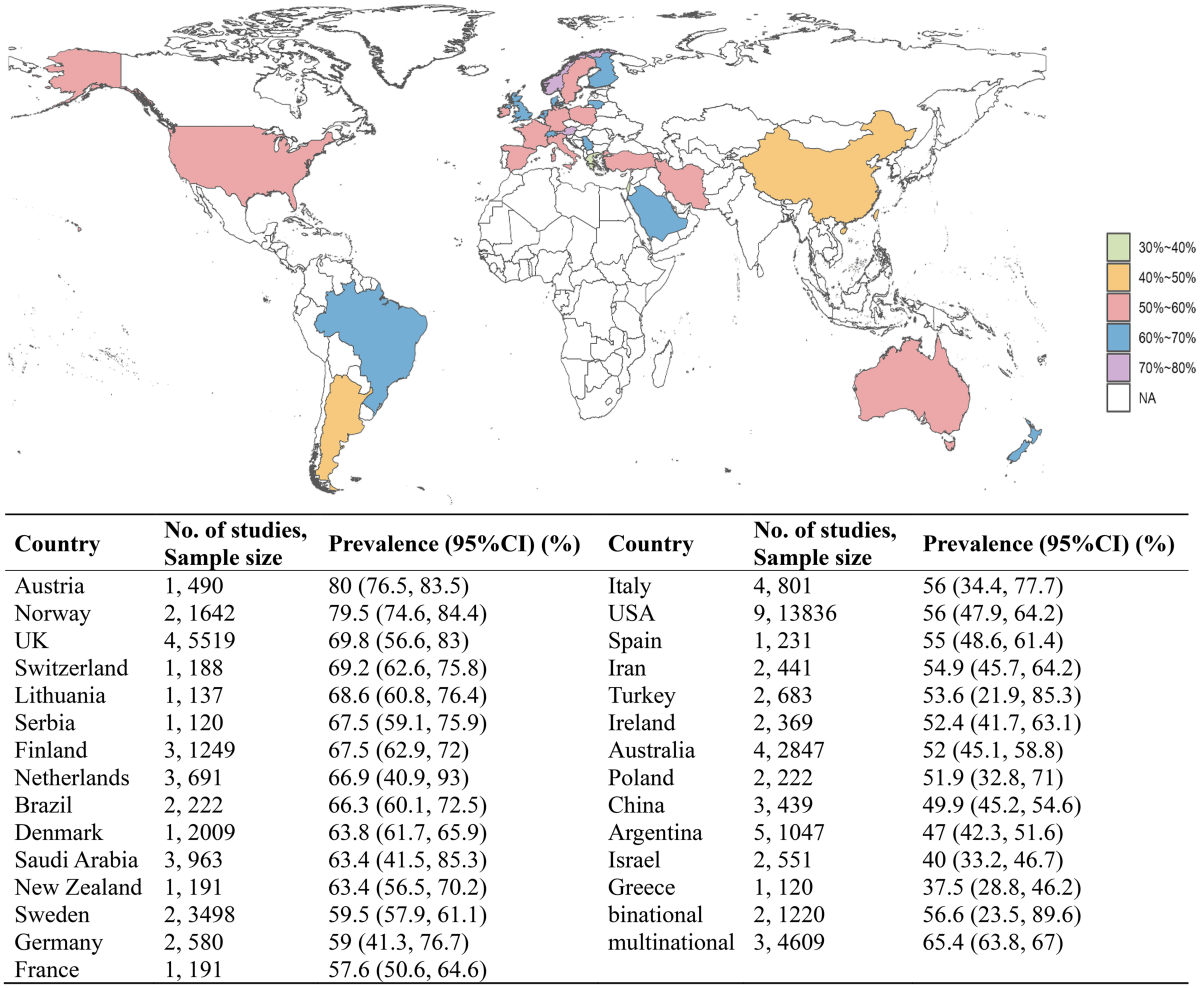
The pooled prevalences of MS-related fatigue by country.

Fatigue estimates ranged from 28.4 to 88.2%. The pooled prevalence of fatigue was 59.1% (95% CI: 55.9–62.2%), with significant heterogeneity (*I*^2^ = 97.3%, *p* < 0.01) (Supplementary Figure 1). Several studies additionally reported prevalence rates by sex, MS phenotype, and education level, and we pooled these results as well. In 15 studies, the prevalence of 6,982 females was greater than that of 2,598 males (58 and 56.5%, respectively). For the prevalence by MS phenotype, SPMS had the highest prevalence (74.4%, *n* = 10 studies), followed by PPMS (64.3%, *n* = 8 studies) and RRMS (54.7%, *n* = 15 studies). The prevalence among individuals with >12 years of education (47.9%) was lower than that of individuals with ≤12 years of education (64.3%) among the 4 studies. Additional details of the results are provided in Supplementary Table 7. The five countries with the highest prevalence of fatigue were Austria (80%), Norway (79.5%), the UK (69.8%), Switzerland (69.2%) and Lithuania (68.6%). The prevalences in the remaining countries and the global prevalence distribution are shown in Figure 2.

After excluding studies with a considerable risk of selection or attrition bias, the estimates for the prevalence of fatigue remained largely unchanged. Overall, the sensitivity analysis indicated that the findings were robust and reliable (Supplementary Figure 2).

### 3.6 Subgroup analysis

We regrouped the included studies based on study and sample characteristics for subgroup analyses to explore the high heterogeneity. Table 1 shows the results of the subgroup meta-analyses. Subgroup analysis by sample size showed that the pooled prevalence of the studies with sample sizes >1000 was greater than that of the studies with sample sizes <1000, at 67 and 59.9%, respectively. The prevalence decreased over the survey years, from 64.4% in 2000 ~ 2009 to 59.6% in 2010 ~ 2019 and to 51% in 2020 ~ 2023. Prevalence increased with the proportion of females, from 49.9% (<60%) to 58.5% (60–80%) and then to 64.6% (≥80%). The results for the three age strata (30–40 years, 40–50 years, 50–60 years) revealed an increasing prevalence of fatigue among PwMS, with rates of 54.7, 58.6, and 69.3%, respectively. According to the subgroup analysis by EDSS score, the prevalence was greater in PwMS with scores >4 than in those with scores ≤4, reaching 73.7%. For MS duration, 63.4% of patients with an MS duration >10 years had a higher prevalence of MS-related fatigue at 63.4% compared to those with an MS duration ≤10 years (55.6%). Regarding the prevalence measured by different fatigue scales, the FSMC produced the highest estimate (70.4%), the MFIS, with a cut-off of 38, had the lowest (51%), and the most common scale was the FSS, where the prevalence of fatigue with a reference cut-off value of 4 was greater than that with a reference cut-off value of 5, standing at 65.6 and 54.3%, respectively. Among patient source subgroups, the pooled prevalence was 61.4% in population-based studies, 58.9% in outpatient studies, 50.2% in MS Medical Research Centres, and 54.9% in mixed population studies. When assessed by WHO region, the EUR had the highest estimate of fatigue (61.2%), and the WPR had the lowest estimate of fatigue (54.2%). Moreover, the estimated pooled prevalence was significantly greater in high-income countries (59.9%) than in upper-middle-income countries (53.4%). Of the 10 study and sample characteristics for which subgroup analyses were conducted, all but the WHO region and patient source had a statistically significant impact on study reported prevalence of fatigue, suggesting that these factors are potential sources of heterogeneity between studies.

**Table 1:** Subgroup analyses of the prevalence of fatigue in MS patients.

Subgroup	No. of studies	Sample size	Prevalence (95% CI) (%)	*I*^2^(%)	Heterogeneity between groups *p* value
Sample size					
<1000	60	14952	59.9 (59.2–60.7)	96.1	
>1000	9	29516	67 (66.5–67.6)	98.9	0.0251*
Survey year					
2000 ~ 2009	12	12083	64.4 (58.4–70.4)	95.5	
2010 ~ 2019	46	25136	59.6 (55.6–63.6)	96.5	
2020 ~ 2023	11	7249	51 (44.5–57.5)	97.2	0.011*
Patient source					
Population-based	34	37929	61.4 (56.9–65.8)	98.2	
MS Medical Research Centre	4	484	50.2 (23.9–76.6)	98.3	
Outpatient	24	4740	58.9 (54.9–62.9)	88.1	
Mixed	7	1517	54.9 (46.4–63.4)	90	0.5045
Age (years)					
30 ~ 40	16	2778	54.7 (49.4–60)	88.8	
40 ~ 50	40	31265	58.6 (54.5–62.6)	97.2	
50 ~ 60	10	8866	69.3 (63.8–74.8)	97.3	0.0004*
Proportion of females					
<60%	3	467	49.9 (45.4–54.4)	37	
60 ~ 80%	55	32925	58.5 (54.9–62.3)	97.5	
≥80%	11	10842	64.6 (58.1–71.1)	94.4	0.0005*
MS duration					
≤10 years	22	9666	55.6 (49.9–61.3)	95.9	
>10 years	21	23062	63.4 (58.1–68.7)	96.9	0.0479*
EDSS score					
≤4	21	7546	56 (49.9–62.1)	95.3	
>4	4	5049	73.7 (65.8–81.5)	96.8	0.0005*
Fatigue scale					
FSS cutoff 4	28	25969	65.6 (61.1–70)	97.1	
FSS cutoff 5	17	8800	54.3 (50–58.6)	92.2	
MFIS cutoff 38	14	3253	48.8 (41.4–56.3)	97.5	
FSMC	4	5125	70.4 (61–79.9)	98.9	<0.0001*
WHO region					
AMR	16	15105	54.5 (49–60.1)	98.3	
EMR	5	1404	60.2 (47.1–73.4)	98	
EUR	36	19291	61.2 (56.3–66.1)	97	
WPR	9	4227	54.2 (49.6–58.8)	80.4	0.1526
Income level					
Upper-middle	15	2952	53.4 (48.2–58.6)	88.8	
High	53	37657	59.9 (56.1–63.6)	97.5	0.049*

Subgroup analyses were performed for variables examined in at least three studies. AMR, Region of the Americas; CI, confidence interval; EDSS, Extended Disability Status Scale; EUR, European Region; EMR, Eastern Mediterranean Region; FSMC, Fatigue Scale for Motor and Cognitive Functions; FSS, Fatigue Severity Scale; HI, high income; MFIS, Modified Fatigue Impact Scale; WHO, World Health Organization; WPR, Western Pacific Region. *p < 0.05.

### 3.7 Meta-regression analysis

Although subgroup analyses were performed to assess sources of heterogeneity, large variation between studies was still present within each subgroup. To further clarify and explain the sources of heterogeneity, we performed meta-regression analyses. The findings indicated that age, fatigue scale, MS duration, and EDSS score were significantly correlated with heterogeneity, explaining 14.6, 46.4, 7.1, and 18.4% of the between-study variance, respectively, for a total of 86.4%. The meta-regression analysis results are shown in Table 2.

**Table 2:** Results of the meta-regression analyses.

Variable	Coef.	SE	*p* value	Adj *R*^2^ (%)
Sample size			0.136	2.23
<1000	reference			
>1000	0.0712	0.0477	0.136	
Survey year			0.116	4.17
2000 ~ 2009	reference			
2010 ~ 2019	−0.0656	0.0446	0.142	
2020 ~ 2023	−0.1334	0.0658	0.043	
Patient source			0.273	1.57
Academic MS Medical Research Centre	reference			
Population-based	0.1168	0.0699	0.095	
Outpatient	0.0657	0.0712	0.357	
Mixed	0.0649	0.0875	0.459	
Age (years)			0.005*	14.61
30 ~ 40	reference			
40 ~ 50	0.0502	0.0400	0.209	
50 ~ 60	0.1603	0.0506	0.002	
Proportion of females			0.311	0.69
<60%	reference			
60 ~ 80%	0.0867	0.0785	0.270	
≥80%	0.1307	0.0872	0.134	
MS duration			0.049*	7.05
≤10 years	reference			
>10 years	0.0778	0.0396	0.049	
EDSS score			0.014*	18.38
≤4	reference			
>4	0.1786	0.0727	0.014	
Fatigue tool			<0.001*	46.44
FSMC	reference			
FSS cutoff 4	−0.0376	0.0510	0.461	
FSS cutoff 5	−0.1679	0.0535	0.002	
MFIS cutoff 38	−0.2405	0.0554	0.0001	
WHO region			0.174	3.94
AMR	reference			
EMR	0.0494	0.1332	0.711	
EUR	0.0737	0.0409	0.072	
WPR	−0.0095	0.0547	0.863	
Income level			0.113	3.44
High	reference			
Upper-middle	−0.0666	0.0420	0.113	

*p < 0.05.

## 4 Discussion

We identified 69 studies from 27 countries that assessed the prevalence of fatigue in PwMS from 2000 to the present. A meta-analysis revealed that 59.1% of PwMS worldwide suffer from fatigue. Significant heterogeneity was detected, which could be explained by the fatigue scale, age, EDSS score, and MS duration, with the fatigue scale being the largest source of heterogeneity. Although these variables explained approximately 86% of the variance, the heterogeneity remained significant, so the combined results still should be treated with appropriate caution.

A systematic literature review in 2021 reported that the prevalence of fatigue in PwMS ranged from 36.5 to 78%, and our estimated global prevalence of 59.1% is close to the median prevalence rate of this range, which is 57.25%. Given that the prevalence of fatigue in the general adult population is 20.4% (35), the prevalence of fatigue in individuals with MS is quite high. Notably, we found that the prevalence of fatigue in PwMS exceeded the prevalence in patients with other neurological diseases by a minimum of 9%. Siciliano et al. conducted a meta-analysis and reported a 50% prevalence of fatigue among patients with Parkinson’s disease (36). Zhan et al. estimated a global prevalence of fatigue after stroke of 46.79% (37). Hamad et al.’s meta-analysis showed that the prevalence of fatigue in amyotrophic lateral sclerosis (ALS) patients was 48% (38). Drawing upon the most recent Multiple Sclerosis Atlas, a collaborative effort between the Multiple Sclerosis International Federation and the WHO, the global tally of individuals affected by multiple sclerosis stands at 2.8 million (2, 3). We estimate that approximately 1.65 million PwMS around the world are affected by fatigue. It is important to recognize that 59.1% of all PwMS experience clinically significant fatigue; therefore, they are potential candidates who seek fatigue management and treatment and should be given priority. Owing to the subjective nature of fatigue manifestations, symptoms of fatigue are often clinically overlooked during MS treatment (1, 10). It is only within the past decade, the impact of MS fatigue on daily life has gradually been recognized as an important aspect of clinical practice and research (12). More than two-thirds of the studies included in our systematic review were conducted from 2014 to the present. Thus, fatigue assessment should become a mandatory clinical procedure to ensure that this symptom is not missed in the clinical workup and is appropriately addressed within the subsequent treatment program. Regarding the prevalence in different WHO regions, the subgroups did not significantly differ from each other. This suggests that the prevalence of MS-related fatigue has cross-regional stability. This finding may be relevant for aetiological research, which should not expect risk factors with a large magnitude of effect to vary across geography. Our study lacked data in two regions around the equator, the AFR and the SEAR. This is related to the epidemiological pattern of MS, which is less prevalent at lower latitudes (2, 7). In our study, subgroup analysis revealed a downwards secular trend in the prevalence of fatigue among PwMS over the past two decades. Specifically, the pooled prevalence of fatigue from 2020 to the present has decreased by 13.4 percentage points compared to that between 2000 and 2009. This considerable reduction was primarily attributed to the sustained efforts of MS professionals over the years in achieving advancements in the treatment and management of MS-related fatigue. Drug-free treatments involving different professionals are currently considered the best strategy for reducing fatigue in MS patients (10). Physical exercise has long been recommended, with aerobic exercise and resistance training being the most effective (39, 40). It also seems promising to improve fatigue by modifying the diet structure, such as through the use of an anti-inflammatory diet (41). Studies have shown that a low-fat vegetarian or Mediterranean diet has the potential to reduce both chronic and acute fatigue in PwMS (42). In addition, psychological interventions (43), including cognitive behavioral therapy (CBT) and mindfulness therapy, also have potential effects.

The included studies used 4 validated fatigue tools, with both the FSS and MFIS reporting at least 3 different cut-off values. Although previous studies have confirmed the reliability and validity of the different assessment scales, these scales have their own features. The FSS evaluates the sole dimension of physical fatigue, responding to the severity, frequency, and impact on one’s daily routine. The other scales all evaluate multiple dimensions of fatigue; the FIS explores the functional limitations (cognitive, physical and psychosocial) caused by fatigue; the MFIS is a shorter version of the FIS; and the FSMC measures motor and cognitive fatigue (9, 21, 44). These differences between the fatigue scales resulted in unwanted variability in measurement across PwMS. In addition, the prevalence of fatigue could be impacted by different cut-off value references, even if the same screening instrument was employed. Subgroup analyses revealed that the prevalence of fatigue in high-income countries was greater (59.9%) than that in upper-middle-income countries. These discrepancies may be largely due to the lack of measurement equivalence. Among upper-middle-income countries, the number of studies using the MFIS, the FSS (cut-off 5) and the FSS (cut-off 4) was almost equal. However, half of the studies conducted in high-income countries used the FSS (cut-off 4). The studies employing the FSMC were all from high-income countries. According to the subgroup analysis, a higher prevalence was reported using these two instruments, the FSMC and the FSS (cut-off 4). In light of these results, overcoming heterogeneity requires the systematic use of uniform measures to define fatigue in MS patients. Currently, the FSS and MFIS seem to be considered the gold standards for assessing fatigue in PwMS (10). Nevertheless, in our study, the prevalence of fatigue estimated by these two scales differed by a staggering 11.3% (FSS 65.1% vs. MFIS 53.9%). Recently, the Academy of Neurologic Physical Therapy (ANPT) assigned the MS Outcome Measures Task Force to conduct a systematic review of self-reported fatigue measures used for PwMS. Ultimately, the Task Force recommended that clinicians and researchers utilize the MFIS for comprehensive measurement or the FSS for screening (21). MS-related fatigue, as a group of self-reported symptoms, is often complex and multidimensional, and the use of the FSS, which focuses on a single dimension of fatigue, is somewhat limited in its use as a screening tool. A study comparing the validity and responsiveness of a questionnaire called the PROMIS Fatigue (MS) 8a with these two scales revealed that the PROMIS Fatigue (MS) 8a performed better (45). Additionally, the optimal and suitable cut-off values for the FSS and MFIS are still uncertain. Therefore, further research needs to be conducted in the future to develop methodological guidelines or consensus on the best tools for screening MS-related fatigue to more accurately determine its prevalence.

Meta-regression analysis showed that age could explain 14.6% of the heterogeneity among the prevalence rates, and the older subgroup reported more fatigue. It may be a result of health comorbidities or deconditioning that accompanies older age. The global average age at multiple sclerosis diagnosis is estimated to be 32 years (2), which is in early adulthood, meaning that older PwMS typically suffer from a longer MS duration. Our results showed that MS duration was also a marked moderator of the fatigue prevalence, with a significantly greater prevalence in those with a duration of more than 10 years than in those with a duration of 10 years or less (63.4% vs. 55.6%). Fatigue was present in 35.3% ~ 46% of patients at the time of the first attack of MS (46, 47). A study from Canada reported an incidence of fatigue of 28.9% per 100 PwMS in the first year after enrolment, 29.9% in the second year, and an overall cumulative incidence of 38.8% (48). Thus, fatigue is not only present early in MS but may also be a consequence of MS duration (49, 50). Healthcare professionals should screen and diagnose fatigue in patients at the early stage of MS onset, intervene early and establish a longitudinal follow-up system to persevere PwMS’s employment and HRQoL (6, 51) and more accurately assess the prevalence of MS-related fatigue.

Sex differences in the prevalence of MS-related fatigue between studies are contradictory. Some studies found that fatigue was sex-related and prevalent in females (18, 43, 52), while others did not observe significant differences in prevalence between males and females (50, 53, 54). Additionally, some studies have shown that females are more prone to cognitive fatigue (52, 55). According to our study, females exhibited a greater prevalence of fatigue than males, and subgroup analyses also revealed that studies with a greater proportion of females reported a greater prevalence of fatigue. Therefore, our findings support the notion that fatigue is more prevalent among females. The evidence suggests that females are more likely to suffer from sleep disorders (56, 57) and depression (58), yet in PwMS, these symptoms can lead to secondary fatigue (9, 10, 50, 59). Furthermore, females are typically diagnosed with MS during their childbearing years (7), a period in which they shoulder the long-term and arduous responsibilities of childbirth and raising and caring for children. Research has also indicated that MS females who have children experience a greater level of fatigue (16, 43). Females who are experiencing perimenopause or menopause may experience endocrine dysfunction, abnormal hormone metabolism, and other issues, which can subsequently lead to mental health problems such as chronic fatigue, anxiety, and depression (60). Therefore, healthcare professionals should pay sufficient attention to the mental health status of female PwMS, conduct early screening and evaluation, and encourage family members to actively participate in the treatment and management of MS to enhance patients’ perceptions and utilization of family support.

Our research showed that PwMS with higher education levels reported less fatigue (47.9% vs. 64.3%). This discovery confirmed the findings of Kroencke et al. who evaluated 540 PwMS in a community-based setting and observed that PwMS with higher education levels consistently reported lower levels of fatigue (61). Evidence indicates that PwMS with lower education levels are more likely to adopt health risk behaviors, including unhealthy eating habits, a sedentary lifestyle, smoking, etc. (62). In large international longitudinal studies, these behaviors were identified as absolute risk factors for clinically significant fatigue in PwMS (63). In contrast, PwMS with higher education generally have greater health literacy and the ability to cope with fatigue (16), including actively seeking information on disease prevention and treatment through internet platforms (43), adhering to a healthy lifestyle, or actively participating in healthcare. Furthermore, PwMS with higher education levels tend to have greater cognitive reserve (64) and are less likely to experience cognitive fatigue. One study pooled the results of 10 RCTs focusing on educational intervention programs, and the evidence showed that these educational programs could effectively alleviate MS-related fatigue and have long-term effects (65). Therefore, education has a protective effect on MS-related fatigue, and healthcare professionals can improve patients’ health literacy and correct modifiable unhealthy lifestyles by strengthening their education on disease and fatigue management.

Previous studies confirmed that disability is independently associated with fatigue in MS (8, 14, 66). Similarly, we found that disability status was a significant modifier of the prevalence of fatigue among PwMS. The prevalence of fatigue in PwMS with an EDSS score greater than 4 was 17.7% greater than that in patients with an EDSS score of 4 or less. Moreover, it seems that a significantly greater proportion of PwMS with higher EDSS scores experienced severe fatigue (18). Although the exact pathophysiological mechanisms of fatigue remain incompletely understood (10), a study suggested that fatigue and disability may share underlying mechanisms in MS (13). Interestingly, we observed that the prevalence of fatigue among the three MS phenotypes exhibited a 10% gradient: RRMS, 54.7%; PPMS, 64.3%; and SPMS, 74.4%. The disparity in fatigue prevalence among various MS phenotypes could primarily stem from the divergence in disability status (13, 67), as patients with progressive MS tended to experience more fatigue due to their greater degree of disability.

We further investigated other methodological factors by applying some eligibility criteria. Regarding patient recruitment resources, although subgroup analysis revealed that fatigue was more prevalent in population-based and outpatient patients than in Academic MS Medical Research Centre and mixed-source patients, the difference was not significant. In terms of the sample size, there was no evidence showing a significant difference in MS-related fatigue between studies with a sample size greater than 1,000 and those with a sample size less than 1,000, which could be explained by the fact that fatigue is prevalent in MS, as it is distinct from rare diseases that require a larger sample size for identification.

### 4.1 Strengths and limitations

To the best of our knowledge, an estimation of the prevalence of fatigue in PwMS worldwide has not been performed to date. This is the first meta-analysis to estimate the worldwide pooled prevalence of fatigue in PwMS and the first attempt to account for the observed high heterogeneity. Our study synthesized data on MS-related fatigue from 27 countries across 4 WHO regions and may provide useful information for clarifying the relationship between fatigue and the characteristics of PwMS. To control for methodological bias, we included studies that used validated fatigue scales and explicitly reported the cutoff values adopted. We excluded hospitalized patients and aimed to define the pooled prevalence of fatigue in as representative a global population of PwMS as possible. These results have important implications to healthcare, research planning, and policy related to MS around the world.

Several intrinsic limitations of this study should also be recognized. First, we imposed a language limitation and retrieved studies in English and Chinese. Therefore, linguistic bias cannot be excluded. Although we established some inclusion criteria for the studies, we detected substantial heterogeneity, which did not significantly decrease after subgroup analyses. The assessment tools used for MS-related fatigue in the included studies were not unified, which may reduce the reliability of the results due to measurement bias. Additionally, the distribution of data points across WHO regions was uneven, with three-quarters of the studies concentrated in two regions (AMR and EUR), lacking data from the AFR and SEAR. Finally, MS-related fatigue is a very complex and multidimensional condition that involves physical, cognitive, psychosocial, and spiritual factors, which yield a wide range of definitions. For this reason, this meta-analysis only reported the total scores of the scales; no specific dimensions of fatigue were assessed. Given these limitations, our findings require caution in interpretation.

## 5 Conclusion

This systematic review revealed that MS-related fatigue is widespread globally, with an overall prevalence rate of 59.1% and a decreasing trend over time. Fatigue was prevalent among females, those with lower education levels, those who were older, those with greater disability, and those with longer MS durations. Early fatigue screening and management are crucial for PwMS with the above characteristics. The use of different assessment tools may be the main source of heterogeneity in the differences in the prevalence rates of MS-related fatigue between studies. In the future, efforts should be made to develop methodological guidelines or a consensus on the best tools for screening MS-related fatigue and to explore effective management strategies for fatigue in MS.

## Data Availability

The original contributions presented in the study are included in the article/Supplementary material, further inquiries can be directed to the corresponding author.
