# Supplementary material for: Global prevalence of fatigue in patients with multiple sclerosis: a systematic review and meta-analysis
Source: Front Neurol. 2024 Oct 2;15:1457788. doi: 10.3389/fneur.2024.1457788 (PMC11479926; doi:10.3389/fneur.2024.1457788)
Supplement: Supplementary file 1 [file Table_1.docx]

**Supplementary Material:**

**Supplementary Figure 1** Forest plot of the pooled prevalence of fatigue in MS

**Supplementary Figure 2** The results of sensitivity analysis by removing study one by one

**Supplementary Table 1** PRISMA 2020 Checklist

**Supplementary Table 2** Specific strategy of PubMed

**Supplementary Table 3** The complete citation information for the included studies

**Supplementary Table 4** Characteristics of the included studies and their participants

**Supplementary Table 5** Risk of bias assessment of studies included based on the AHRQ tool

**Supplementary Table 6** Risk of bias assessment of studies included based on the NOS tool

**Supplementary Table 7** Prevalence of fatigue and by sex, phenotypes, and education


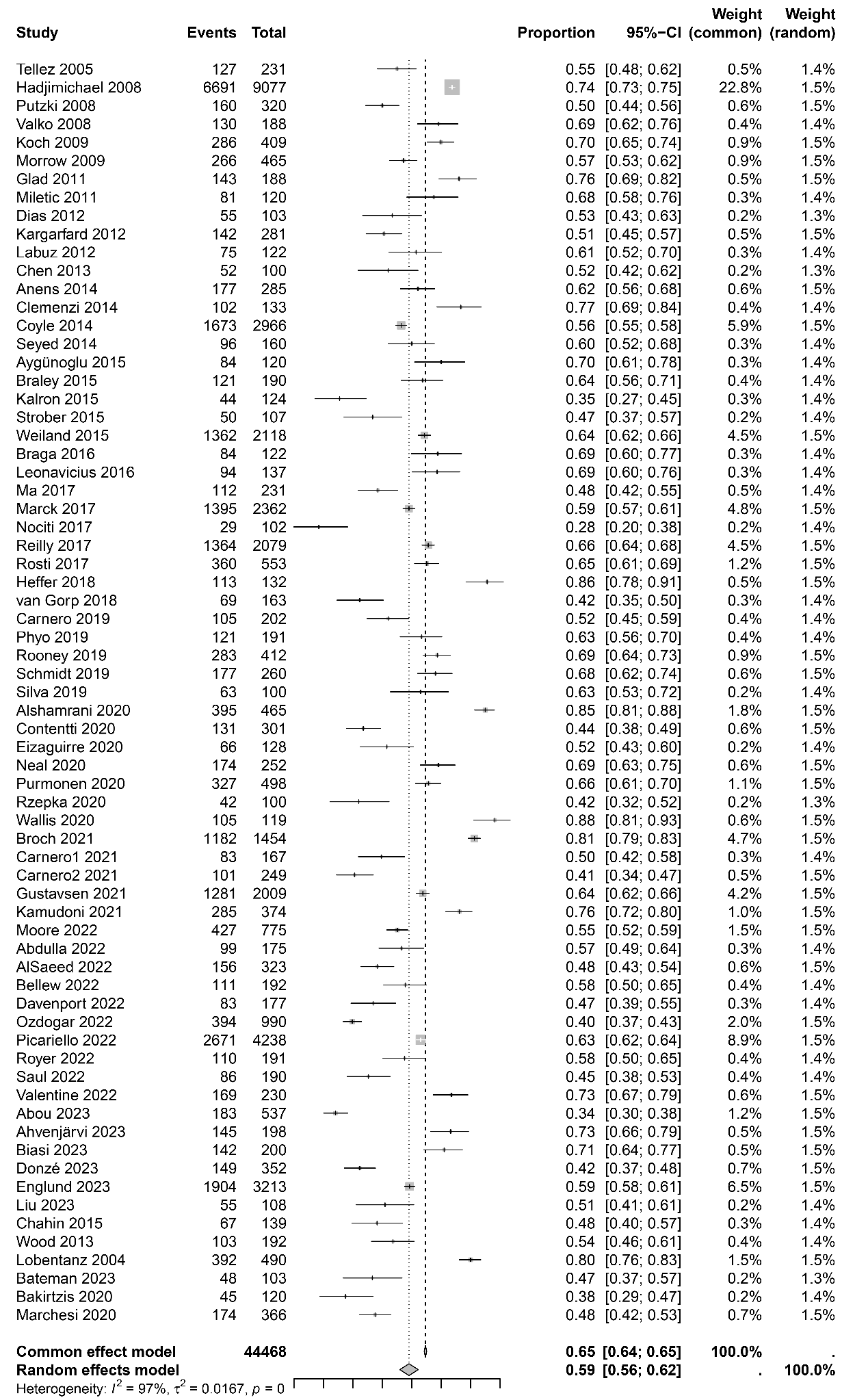


**Supplementary Figure 1** Forest plot of the pooled prevalence of fatigue in MS


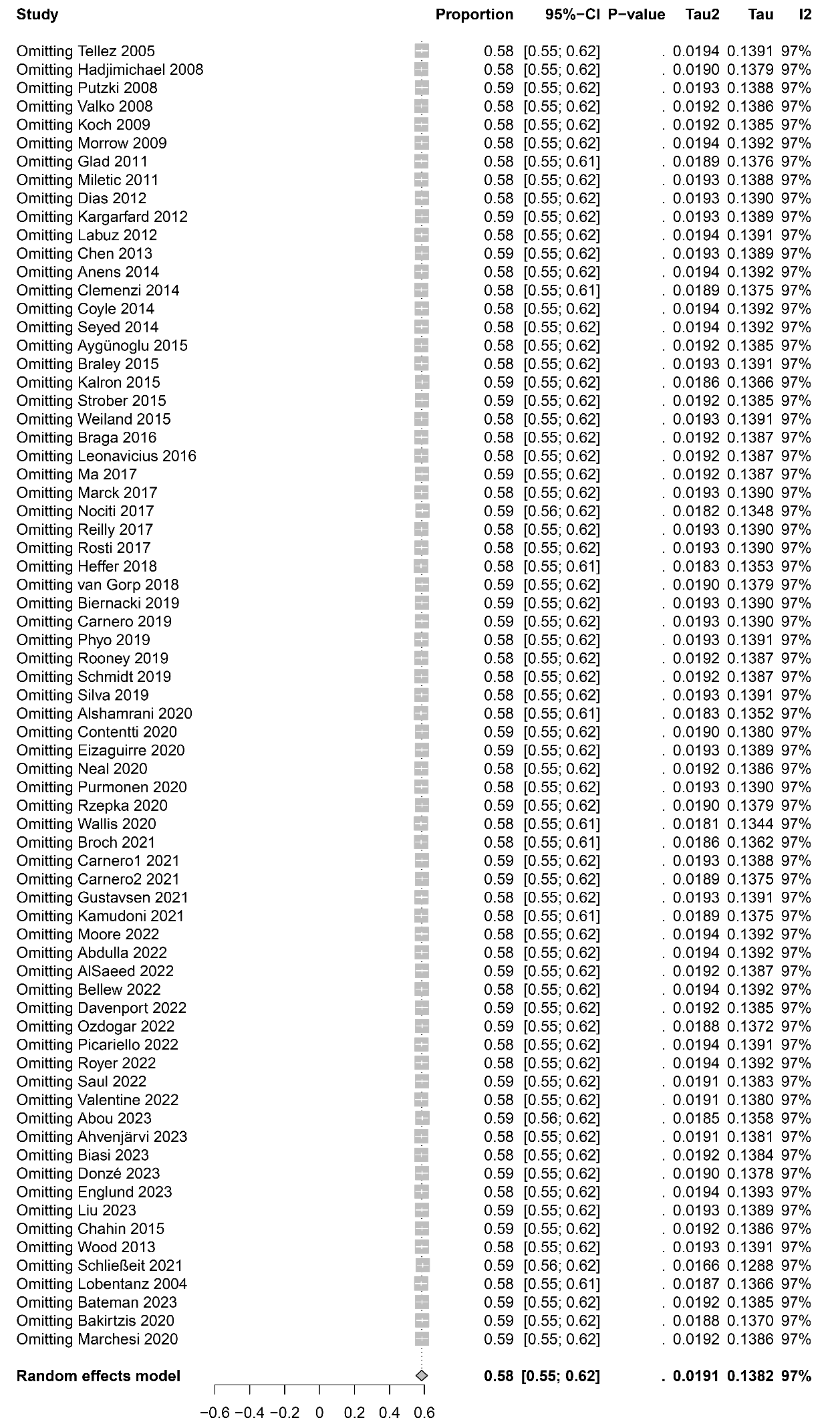


**Supplementary Figure 2** The results of sensitivity analysis by removing study one by one

**Supplementary Table 1** PRISMA 2020 Checklist

| **Section and Topic** | **Item #** | **Checklist item** | **Location where item is reported** |
| --- | --- | --- | --- |
| **TITLE** | | |  |
| Title | 1 | Identify the report as a systematic review. | Title |
| **ABSTRACT** | | |  |
| Abstract | 2 | See the PRISMA 2020 for Abstracts checklist. | Abstract |
| **INTRODUCTION** | | |  |
| Rationale | 3 | Describe the rationale for the review in the context of existing knowledge. | 1 Introduction |
| Objectives | 4 | Provide an explicit statement of the objective(s) or question(s) the review addresses. | 1 Introduction |
| **METHODS** | | |  |
| Eligibility criteria | 5 | Specify the inclusion and exclusion criteria for the review and how studies were grouped for the syntheses. | 2.3 Eligibility criteria |
| Information sources | 6 | Specify all databases, registers, websites, organisations, reference lists and other sources searched or consulted to identify studies. Specify the date when each source was last searched or consulted. | 2.2 Search strategy and data sources |
| Search strategy | 7 | Present the full search strategies for all databases, registers and websites, including any filters and limits used. | Supplementary Table 2 |
| Selection process | 8 | Specify the methods used to decide whether a study met the inclusion criteria of the review, including how many reviewers screened each record and each report retrieved, whether they worked independently, and if applicable, details of automation tools used in the process. | 2.4 Study selection |
| Data collection process | 9 | Specify the methods used to collect data from reports, including how many reviewers collected data from each report, whether they worked independently, any processes for obtaining or confirming data from study investigators, and if applicable, details of automation tools used in the process. | 2.5 Data extraction |
| Data items | 10a | List and define all outcomes for which data were sought. Specify whether all results that were compatible with each outcome domain in each study were sought (e.g. for all measures, time points, analyses), and if not, the methods used to decide which results to collect. | 2.7 Statistical analysis |
|  | 10b | List and define all other variables for which data were sought (e.g. participant and intervention characteristics, funding sources). Describe any assumptions made about any missing or unclear information. | 2.7 Statistical analysis |
| Study risk of bias assessment | 11 | Specify the methods used to assess risk of bias in the included studies, including details of the tool(s) used, how many reviewers assessed each study and whether they worked independently, and if applicable, details of automation tools used in the process. | 2.6 Risk of bias assessment |
| Effect measures | 12 | Specify for each outcome the effect measure(s) (e.g. risk ratio, mean difference) used in the synthesis or presentation of results. | 2.7 Statistical analysis |
| Synthesis methods | 13a | Describe the processes used to decide which studies were eligible for each synthesis (e.g. tabulating the study intervention characteristics and comparing against the planned groups for each synthesis (item #5)). | 2.7 Statistical analysis |
|  | 13b | Describe any methods required to prepare the data for presentation or synthesis, such as handling of missing summary statistics, or data conversions. | 2.7 Statistical analysis |
|  | 13c | Describe any methods used to tabulate or visually display results of individual studies and syntheses. | 2.7 Statistical analysis |
|  | 13d | Describe any methods used to synthesize results and provide a rationale for the choice(s). If meta-analysis was performed, describe the model(s), method(s) to identify the presence and extent of statistical heterogeneity, and software package(s) used. | 2.7 Statistical analysis |
|  | 13e | Describe any methods used to explore possible causes of heterogeneity among study results (e.g. subgroup analysis, meta-regression). | 2.7 Statistical analysis |
|  | 13f | Describe any sensitivity analyses conducted to assess robustness of the synthesized results. | 2.7 Statistical analysis |
| Reporting bias assessment | 14 | Describe any methods used to assess risk of bias due to missing results in a synthesis (arising from reporting biases). | 2.7 Statistical analysis |
| Certainty assessment | 15 | Describe any methods used to assess certainty (or confidence) in the body of evidence for an outcome. | 2.7 Statistical analysis |
| **RESULTS** | | |  |
| Study selection | 16a | Describe the results of the search and selection process, from the number of records identified in the search to the number of studies included in the review, ideally using a flow diagram. | Figure 1 |
|  | 16b | Cite studies that might appear to meet the inclusion criteria, but which were excluded, and explain why they were excluded. | Figure 1 |
| Study characteristics | 17 | Cite each included study and present its characteristics. | Supplementary Table 3 and Supplementary Table 4 |
| Risk of bias in studies | 18 | Present assessments of risk of bias for each included study. | Supplementary Table 5 and Supplementary Table 6 |
| Results of individual studies | 19 | For all outcomes, present, for each study: (a) summary statistics for each group (where appropriate) and (b) an effect estimate and its precision (e.g. confidence/credible interval), ideally using structured tables or plots. | Supplementary Figure 1 |
| Results of syntheses | 20a | For each synthesis, briefly summarise the characteristics and risk of bias among contributing studies. | 3.2 Study and sample characteristics; 3.4 Risk of bias assessment |
|  | 20b | Present results of all statistical syntheses conducted. If meta-analysis was done, present for each the summary estimate and its precision (e.g. confidence/credible interval) and measures of statistical heterogeneity. If comparing groups, describe the direction of the effect. | Supplementary Figure 1; 3.6 Subgroup analysis |
|  | 20c | Present results of all investigations of possible causes of heterogeneity among study results. | 3.6 Subgroup analysis; 3.7 Meta-regression analysis |
|  | 20d | Present results of all sensitivity analyses conducted to assess the robustness of the synthesized results. | 3.6 Subgroup analysis; Supplementary Figure 2 |
| Reporting biases | 21 | Present assessments of risk of bias due to missing results (arising from reporting biases) for each synthesis assessed. | NA |
| Certainty of evidence | 22 | Present assessments of certainty (or confidence) in the body of evidence for each outcome assessed. | NA |
| **DISCUSSION** | | |  |
| Discussion | 23a | Provide a general interpretation of the results in the context of other evidence. | 4 Discussion |
|  | 23b | Discuss any limitations of the evidence included in the review. | 4.1 Strengths and limitations |
|  | 23c | Discuss any limitations of the review processes used. | 4.1 Strengths and limitations |
|  | 23d | Discuss implications of the results for practice, policy, and future research. | 4 Discussion; 4.1 Strengths and limitations |
| **OTHER INFORMATION** | | |  |
| Registration and protocol | 24a | Provide registration information for the review, including register name and registration number, or state that the review was not registered. | 2.1 Protocol and registration |
|  | 24b | Indicate where the review protocol can be accessed, or state that a protocol was not prepared. | 2.1 Protocol and registration |
|  | 24c | Describe and explain any amendments to information provided at registration or in the protocol. | NA |
| Support | 25 | Describe sources of financial or non-financial support for the review, and the role of the funders or sponsors in the review. | Funding |
| Competing interests | 26 | Declare any competing interests of review authors. | Conflict of interest |
| Availability of data, code and other materials | 27 | Report which of the following are publicly available and where they can be found: template data collection forms; data extracted from included studies; data used for all analyses; analytic code; any other materials used in the review. | Supplementary material |

**Supplementary Table 2** Specific strategy of PubMed

| Number | Search detail |
| --- | --- |
| #1 | "Multiple Sclerosis"[MeSH Terms] OR "multiple sclerosis, relapsing remitting"[MeSH Terms] OR "multiple sclerosis, chronic progressive"[MeSH Terms] |
| #2 | "multiple sclerosis, chronic progressive"[MeSH Terms] OR ("multiple"[All Fields] AND "sclerosis"[All Fields] AND "chronic"[All Fields] AND "progressive"[All Fields]) OR "chronic progressive multiple sclerosis"[All Fields] OR ("primary"[All Fields] AND "progressive"[All Fields] AND "multiple"[All Fields] AND "sclerosis"[All Fields]) OR "primary progressive multiple sclerosis"[All Fields] OR ("multiple sclerosis, relapsing remitting"[MeSH Terms] OR ("multiple"[All Fields] AND "sclerosis"[All Fields] AND "relapsing remitting"[All Fields]) OR "relapsing-remitting multiple sclerosis"[All Fields] OR ("relapsing"[All Fields] AND "remitting"[All Fields] AND "multiple"[All Fields] AND "sclerosis"[All Fields]) OR "relapsing remitting multiple sclerosis"[All Fields]) OR ("multiple sclerosis, chronic progressive"[MeSH Terms] OR ("multiple"[All Fields] AND "sclerosis"[All Fields] AND "chronic"[All Fields] AND "progressive"[All Fields]) OR "chronic progressive multiple sclerosis"[All Fields] OR ("secondary"[All Fields] AND "progressive"[All Fields] AND "multiple"[All Fields] AND "sclerosis"[All Fields]) OR "secondary progressive multiple sclerosis"[All Fields]) |
| **#3** | **#1 OR #2** |
| #4 | "Fatigue"[MeSH Terms] |
| #5 | "tried"[All Fields] OR "wear"[Journal] OR "wear"[All Fields] OR "exhaust"[All Fields] OR "exhausts"[All Fields] OR "lackluster"[All Fields] OR "letharg*"[All Fields] OR "fatigue"[MeSH Terms] OR "fatigue"[All Fields] OR "lassitude"[All Fields] |
| **#6** | **#4 OR #5** |
| #7 | "Prevalence"[MeSH Terms] OR "Epidemiology"[MeSH Terms] OR "Epidemiology"[MeSH Subheading] OR "Cross-Sectional Studies"[MeSH Terms] OR "Incidence"[MeSH Terms] OR "Cohort Studies"[MeSH Terms] OR "Case-Control Studies"[MeSH Terms] OR "Epidemiologic Studies"[MeSH Terms] OR "Surveys and Questionnaires"[MeSH Terms] |
| **#8** | **#3 AND #6 AND #7** |

Note. MeSH, Medical Subject Headings.

**Supplementary Table 3** The complete citation information for the included studies

[1] I.S. Lobentanz, S. Asenbaum, K. Vass, C. Sauter, G. Klösch, H. Kollegger, W. Kristoferitsch, J. Zeitlhofer, Factors influencing quality of life in multiple sclerosis patients: Disability, depressive mood, fatigue and sleep quality, Acta Neurol. Scand. 110(1) (2004) 6-13.

[2] T. N, R. J, T. M, N. C, G. I, M. X, Does the Modified Fatigue Impact Scale offer a more comprehensive assessment of fatigue in MS?, Multiple sclerosis: clinical and laboratory research 11(2) (2005) 198-202.

[3] O. Hadjimichael, T. Vollmer, M. Oleen-Burkey, Fatigue characteristics in multiple sclerosis: the North American Research Committee on Multiple Sclerosis (NARCOMS) survey, Health Qual Life Outcomes 6 (2008) 100.

[4] N. Putzki, Z. Katsarava, S. Vago, H.C. Diener, V. Limmroth, Prevalence and severity of multiple-sclerosis-associated fatigue in treated and untreated patients, Eur. Neurol. 59(3-4) (2008) 136-142.

[5] P.O. Valko, C.L. Bassetti, K.E. Bloch, U. Held, C.R. Baumann, Validation of the Fatigue Severity Scale in a Swiss cohort, Sleep: Journal of Sleep and Sleep Disorders Research 31(11) (2008) 1601-1607.

[6] M. Koch, J. Mostert, M. Heerings, M. Uyttenboogaart, J. De Keyser, Fatigue, depression and disability accumulation in multiple sclerosis: A cross-sectional study, Eur. J. Neurol. 16(3) (2009) 348-352.

[7] S.A. Morrow, B. Weinstock-Guttman, F.E. Munschauer, D. Hojnacki, R.H. Benedict, Subjective fatigue is not associated with cognitive impairment in multiple sclerosis: cross-sectional and longitudinal analysis, Multiple sclerosis (Houndmills, Basingstoke, England) 15(8) (2009) 998-1005.

[8] S.B. Glad, H. Nyland, J.H. Aarseth, T. Riise, K.M. Myhr, How long can you keep working with benign multiple sclerosis?, J. Neurol. Neurosurg. Psychiatry 82(1) (2011) 78-82.

[9] S. Miletic, G. Toncev, J. Jevdjic, B. Jovanovic, D. Canovic, FATIGUE AND DEPRESSION IN MULTIPLE SCLEROSIS: CORRELATION WITH QUALITY OF LIFE, Arch. Biol. Sci. 63(3) (2011) 617-622.

[10] R.A. Dias, K.A. Hardin, H. Rose, M.A. Agius, M.L. Apperson, S.D. Brass, Sleepiness, fatigue, and risk of obstructive sleep apnea using the STOP-BANG questionnaire in multiple sclerosis: a pilot study, Sleep & breathing = Schlaf & Atmung 16(4) (2012) 1255-65.

[11] M. Kargarfard, M. Eetemadifar, M. Mehrabi, A.H. Maghzi, M.R. Hayatbakhsh, Fatigue, depression, and health-related quality of life in patients with multiple sclerosis in Isfahan, Iran, Eur. J. Neurol. 19(3) (2012) 431-437.

[12] B. Labuz-Roszak, K. Kubicka-Baczyk, K. Pierzchata, A. Machowska-Majchrzak, M. Skrzypek, Fatigue and its association with sleep disorders, depressive symptoms and anxiety in patients with multiple sclerosis, Neurol. Neurochir. Pol. 46(4) (2012) 309-317.

[13] K. Chen, Y. Fan, R. Hu, T. Yang, K. Li, Impact of depression, fatigue and disability on quality of life in Chinese patients with multiple sclerosis, Stress and health : journal of the International Society for the Investigation of Stress 29(2) (2013) 108-12.

[14] B. Wood, I.A.F. Van Der Mei, A.L. Ponsonby, F. Pittas, S. Quinn, T. Dwyer, R.M. Lucas, B.V. Taylor, Prevalence and concurrence of anxiety, depression and fatigue over time in multiple sclerosis, Mult. Scler. J. 19(2) (2013) 217-224.

[15] E. Anens, M. Emtner, L. Zetterberg, K. Hellström, Physical activity in subjects with multiple sclerosis with focus on gender differences: a survey, BMC neurology 14 (2014) 47.

[16] A. Clemenzi, A. Pompa, P. Casillo, L. Pace, E. Troisi, S. Catani, M.G. Grasso, Chronic pain in multiple sclerosis: is there also fibromyalgia? An observational study, Medical science monitor : international medical journal of experimental and clinical research 20 (2014) 758-66.

[17] P.K. Coyle, B.A. Cohen, T. Leist, C. Markowitz, M. Oleen-Burkey, M. Schwartz, M.J. Tullman, H. Zwibel, Therapy optimization in multiple sclerosis: a prospective observational study of therapy compliance and outcomes, BMC neurology 14 (2014) 9.

[18] S.M. Seyed Saadat, M. Hosseininezhad, B. Bakhshayesh, S.N. Seyed Saadat, S.P. Nabizadeh, Prevalence and predictors of depression in Iranian patients with multiple sclerosis: a population-based study, Neurological sciences : official journal of the Italian Neurological Society and of the Italian Society of Clinical Neurophysiology 35(5) (2014) 735-40.

[19] S.K. Aygünoglu, A. Çelebi, N. Vardar, E. Gürsoy, Correlation of Fatigue with Depression, Disability Level and Quality of Life in Patients with Multiple Sclerosis, Noropsikiyatri Ars. 52(3) (2015) 247-251.

[20] T.J. Braley, B.M. Segal, R.D. Chervin, Hypnotic use and fatigue in multiple sclerosis, Sleep Med 16(1) (2015) 131-7.

[21] S. Chahin, D. Miller, R.E. Sakai, J.A. Wilson, T. Frohman, C. Markowitz, D. Jacobs, A. Green, P.A. Calabresi, E.M. Frohman, S.L. Galetta, L.J. Balcer, Relation of quantitative visual and neurologic outcomes to fatigue in multiple sclerosis, Mult Scler Relat Disord 4(4) (2015) 304-10.

[22] A. Kalron, Association between perceived fatigue and gait parameters measured by an instrumented treadmill in people with multiple sclerosis: a cross-sectional study, J Neuroeng Rehabil 12 (2015) 34.

[23] L.B. Strober, Fatigue in multiple sclerosis: A look at the role of poor sleep, Front. Neurol. 6(FEB) (2015).

[24] T.J. Weiland, G.A. Jelinek, C.H. Marck, E.J. Hadgkiss, D.M. van der Meer, N.G. Pereira, K.L. Taylor, Clinically significant fatigue: prevalence and associated factors in an international sample of adults with multiple sclerosis recruited via the internet, PLoS One 10(2) (2015) e0115541.

[25] D.M. Braga, G.F. Prado, D.B. Bichueti, E.M. Oliveira, Positive correlation between functional disability, excessive daytime sleepiness, and fatigue in relapsing-remitting multiple sclerosis, Arq Neuropsiquiatr 74(6) (2016) 433-8.

[26] R. Leonavicius, Among multiple sclerosis and fatigue, Neurology Psychiatry and Brain Research 22(3-4) (2016) 141-145.

[27] S.L. Ma, X. Rui, P.Y. Qi, G.Q. Liu, J. Yang, Sleep disorders in patients with multiple sclerosis in China, Sleep Breath. 21(1) (2017) 149-154.

[28] C.H. Marck, A.M. De Livera, T.J. Weiland, P.L. Jelinek, S.L. Neate, C.R. Brown, K.L. Taylor, F. Khan, G.A. Jelinek, Pain in People with Multiple sclerosis: associations with Modifiable lifestyle Factors, Fatigue, Depression, anxiety, and Mental health Quality of life, Front. Neurol. 8 (2017) 7.

[29] V. Nociti, F.A. Losavio, V. Gnoni, A. Losurdo, E. Testani, C. Vollono, G. Frisullo, V. Brunetti, M. Mirabella, G. Della Marca, Sleep and fatigue in multiple sclerosis: A questionnaire-based, cross-sectional, cohort study, J Neurol Sci 372 (2017) 387-392.

[30] G.D. Reilly, A.S. Mahkawnghta, P.L. Jelinek, A.M. De Livera, T.J. Weiland, C.R. Brown, K.L. Taylor, S.L. Neate, G.A. Jelinek, C.H. Marck, International Differences in Multiple Sclerosis Health Outcomes and Associated Factors in a Cross-sectional Survey, Front. Neurol. 8 (2017) 10.

[31] E. Rosti-Otajärvi, P. Hämäläinen, A. Wiksten, T. Hakkarainen, J. Ruutiainen, Validity and reliability of the Fatigue Severity Scale in Finnish multiple sclerosis patients, Brain Behav. 7(7) (2017) 8.

[32] P. Heffer-Rahn, P.L. Fisher, The clinical utility of metacognitive beliefs and processes in emotional distress in people with multiple sclerosis, J Psychosom Res 104 (2018) 88-94.

[33] D.A.M. van Gorp, J.J.L. van der Klink, F.I. Abma, P.J. Jongen, I. van Lieshout, E.P.J. Arnoldus, E.A.C. Beenakker, H.M. Bos, J.J.J. van Eijk, J. Fermont, S.T.F.M. Frequin, K. de Gans, G.J.D. Hengstman, R.M.M. Hupperts, J.P. Mostert, P.H.M. Pop, W.I.M. Verhagen, D. Zemel, M.A.P. Heerings, M.F. Reneman, H.A.M. Middelkoop, L.H. Visser, K. van der Hiele, The capability set for work - correlates of sustainable employability in workers with multiple sclerosis, Health Qual. Life Outcomes 16(1) (2018).

[34] E. Carnero Contentti, J.P. Pettinicchi, A. Caride, P.A. López, Sexual Dysfunction in Patients with Multiple Sclerosis from Argentina: What are the Differences Between Women and Men?, Sexuality & Disability 37(4) (2019) 521-539.

[35] A.Z.Z. Phyo, G.A. Jelinek, C.R. Brown, E. O'Kearney, S.L. Neate, A.M. De Livera, K.L. Taylor, W. Bevens, S. Simpson, T.J. Weiland, Differential multiple sclerosis treatment allocation between Australia and New Zealand associated with clinical outcomes but not mood or quality of life, Mult. Scler. Relat. Disord. 30 (2019) 25-32.

[36] S. Rooney, L. Wood, F. Moffat, L. Paul, Prevalence of fatigue and its association with clinical features in progressive and non-progressive forms of Multiple Sclerosis, Mult Scler Relat Disord 28 (2019) 276-282.

[37] S. Schmidt, P. Jöstingmeyer, Depression, fatigue and disability are independently associated with quality of life in patients with multiple Sclerosis: Results of a cross-sectional study, Mult. Scler. Relat. Disord. 35 (2019) 262-269.

[38] M.d.C.N.d. Silva, D.B.A. Cavalcanti, Evaluation of quality of life in multiple sclerosis patients: impact of fatigue, anxiety and depression, Fisioterapia e Pesquisa 26(4) (2019) 339-345.

[39] F.J. Alshamrani, M.F. Almuaigel, F.A. Alkhamis, A.A. Alsulaiman, N.M. AlMohish, A.F. Albuhassah, A.S. AlZahrani, A.A.M. Zaher, Impact of depression and fatigue on relapsing remitting multiple sclerosis in Kingdom of Saudi Arabia, Saudi Med. J. 41(3) (2020) 290-295.

[40] C. Bakirtzis, I. Nikolaidis, M.K. Boziki, A. Artemiadis, A. Andravizou, L. Messinis, P. Ioannidis, N. Grigoriadis, Cognitive Fatigability is Independent of Subjective Cognitive Fatigue and Mood in Multiple Sclerosis, Cogn. Behav. Neurol. 33(2) (2020) 113-121.

[41] E.C. Contentti, P.A. López, J.P. Pettinicchi, R. Alonso, S. Tizio, V. Tkachuk, A. Caride, I. Galea, Do people with multiple sclerosis want to discuss their long-term prognosis? A nationwide study in Argentina, Mult. Scler. Relat. Disord. 37 (2020) 6.

[42] M.B. Eizaguirre, N. Ciufia, M.S. Roman, C. Martínez Canyazo, R. Alonso, B. Silva, C. Pita, O. Garcea, S. Vanotti, Perceived fatigue in multiple sclerosis: The importance of highlighting its impact on quality of life, social network and cognition, Clin Neurol Neurosurg 199 (2020) 106265.

[43] O. Marchesi, C. Vizzino, A. Meani, L. Conti, G.C. Riccitelli, P. Preziosa, M. Filippi, M.A. Rocca, Fatigue in multiple sclerosis patients with different clinical phenotypes: a clinical and magnetic resonance imaging study, Eur J Neurol 27(12) (2020) 2549-2560.

[44] W.N. Neal, K.L. Cederberg, B. Jeng, J.E. Sasaki, R.W. Motl, Is Symptomatic Fatigue Associated With Physical Activity and Sedentary Behaviors Among Persons With Multiple Sclerosis?, Neurorehabil Neural Repair 34(6) (2020) 505-511.

[45] T. Purmonen, T. Hakkarainen, M. Tervomaa, J. Ruutiainen, Impact of multiple sclerosis phenotypes on burden of disease in Finland, J. Med. Econ. 23(2) (2020) 156-165.

[46] M. Rzepka, M. Toś, M. Boroń, K. Gibas, E. Krzystanek, Relationship between Fatigue and Physical Activity in a Polish Cohort of Multiple Sclerosis Patients, Medicina (Kaunas, Lithuania) 56(12) (2020).

[47] O. Wallis, Y. Bol, S. Köhler, C. van Heugten, Anxiety in multiple sclerosis is related to depressive symptoms and cognitive complaints, Acta Neurol. Scand. 141(3) (2020) 212-218.

[48] L. Broch, C.S. Simonsen, H.Ã. Flemmen, P. Berg-Hansen, Ã. Skardhamar, H. Ormstad, E.G. Celius, High prevalence of fatigue in contemporary patients with multiple sclerosis, Multiple Sclerosis Journal - Experimental, Translational and Clinical 7(1) (2021).

[49] E. Carnero Contentti, P.A. LÃ³pez, J.P. Pettinicchi, V. Tkachuk, M.E. Balbuena, A. Caride, Employment status in people with relapsing multiple sclerosis from Argentina: Impact of disability and neuropsychological factors, Work (Reading, Mass.) 68(4) (2021) 1171-1177.

[50] E. Carnero Contentti, P.A. López, R. Alonso, B. Eizaguirre, J.P. Pettinicchi, S. Tizio, V. Tkachuk, A. Caride, Coping strategies used by patients with relapsing multiple sclerosis from Argentina: correlation with quality of life and clinical features, Neurol Res 43(2) (2021) 126-132.

[51] S. Gustavsen, A. Olsson, H.B. Søndergaard, S.R. Andresen, P.S. Sørensen, F. Sellebjerg, A. Oturai, The association of selected multiple sclerosis symptoms with disability and quality of life: a large Danish self-report survey, BMC neurology 21(1) (2021) 317.

[52] P. Kamudoni, J. Johns, K.F. Cook, R. Salem, S. Salek, J. Raab, R. Middleton, C. Henke, P. Repovic, K. Alschuler, G. von Geldern, A. Wundes, D. Amtmann, Standardizing fatigue measurement in multiple sclerosis: the validity, responsiveness and score interpretation of the PROMIS SF v1.0-Fatigue (MS) 8a, Mult. Scler. Relat. Disord. 54 (2021) 9.

[53] F.A. Abdulla, F.M. Albagmi, F.A. Al-Khamis, Factors that influence quality of life in patients with multiple sclerosis in Saudi Arabia, Disabil. Rehabil. 44(17) (2022) 4775-4783.

[54] S. AlSaeed, T. Aljouee, N.M. Alkhawajah, R. Alarieh, H. AlGarni, S. Aljarallah, M. Ayyash, A. Abu-Shaheen, Fatigue, Depression, and Anxiety Among Ambulating Multiple Sclerosis Patients, Front Immunol 13 (2022) 844461.

[55] D. Bellew, L. Davenport, R. Monaghan, C. Cogley, M. Gaughan, S.M. Yap, N. Tubridy, J. Bramham, C. McGuigan, F. O'Keeffe, Interpreting the clinical importance of the relationship between subjective fatigue and cognitive impairment in multiple sclerosis (MS): How BICAMS performance is affected by MS-related fatigue, Mult. Scler. Relat. Disord. 67 (2022) 7.

[56] L. Davenport, C. Cogley, R. Monaghan, M. Gaughan, M. Yap, J. Bramham, N. Tubridy, C. McGuigan, F. O'Keeffe, Investigating the association of mood and fatigue with objective and subjective cognitive impairment in multiple sclerosis, J. Neuropsychol. 16(3) (2022) 537-554.

[57] H. Moore, K.P.S. Nair, K. Baster, R. Middleton, D. Paling, B. Sharrack, Fatigue in multiple sclerosis: A UK MS-register based study, Mult Scler Relat Disord 64 (2022) 103954.

[58] A.T. Ozdogar, T. Kahraman, S. Ozakbas, A. Achiron, A. Kalron, Fatigue is associated with physical inactivity in people with multiple sclerosis despite different environmental backgrounds: Merging and comparing cohorts from Turkey and Israel, Mult Scler Relat Disord 57 (2022) 103456.

[59] F. Picariello, J. Freeman, R. Moss-Morris, Defining routine fatigue care in Multiple Sclerosis in the United Kingdom: What treatments are offered and who gets them?, Multiple Sclerosis Journal - Experimental, Translational and Clinical 8(1) (2022).

[60] N. Royer, M. Duboeuf, J.-P. Camdessanché, G.Y. Millet, Prevalence of fatigue and its explicative variables among people with multiple sclerosis, Neuro Rehabilitation 51(3) (2022) 509-517.

[61] A. Saul, B.V. Taylor, L. Blizzard, S. Simpson-Yap, W.H. Oddy, Y.C. Probst, L.J. Black, A.L. Ponsonby, S.A. Broadley, J. Lechner-Scott, I. van der Mei, Associations between diet quality and depression, anxiety, and fatigue in multiple sclerosis, Mult Scler Relat Disord 63 (2022) 103910.

[62] T.R. Valentine, K.N. Alschuler, D.M. Ehde, A.L. Kratz, Prevalence, co-occurrence, and trajectories of pain, fatigue, depression, and anxiety in the year following multiple sclerosis diagnosis, Multiple sclerosis (Houndmills, Basingstoke, England) 28(4) (2022) 620-631.

[63] L. Abou, C. McCloskey, C. Wernimont, N.E. Fritz, A.L. Kratz, Examination of Risk Factors Associated With Falls and Injurious Falls in People With Multiple Sclerosis: An Updated Nationwide Study, Arch. Phys. Med. Rehabil. (2023).

[64] H. Ahvenjärvi, M. Niiranen, S. Simula, P. Hämäläinen, H.M. Surcel, A.M. Remes, M. Ryytty, J. Krüger, Fatigue and health-related quality of life depend on the disability status and clinical course in RRMS, Mult. Scler. Relat. Disord. 77 (2023) 8.

[65] G.A. Bateman, A.R. Bateman, J. Lechner-Scott, Dilatation of the bridging cerebral veins in multiple sclerosis correlates with fatigue and suggests an increase in pressure, Mult. Scler. Relat. Disord. 76 (2023) 8.

[66] M.M. Biasi, A. Manni, I. Pepe, C. Abbatantuono, D. Gasparre, P. Iaffaldano, M. Simone, M.F. De Caro, M. Trojano, P. Taurisano, D. Paolicelli, Impact of depression on the perception of fatigue and information processing speed in a cohort of multiple sclerosis patients, BMC Psychol. 11(1) (2023) 7.

[67] C. Donzé, C. Massot, G. Defer, P. Vermersch, P. Lecoz, O. Derepeer, A. Abdullatif, V. Neuville, P. Devos, B. Lennei, M.A. Guyot, L. Norberciackj, P. Hautecoeur, NUTRISEP: Assessment of the nutritional status of patients with multiple sclerosis and link to fatigue, Rev. Neurol. 179(4) (2023) 282-288.

[68] S. Englund, M. Kierkegaard, J. Burman, K. Fink, A. Fogdell-Hahn, M. Gunnarsson, J. Hillert, A. Langer-Gould, J. Lycke, P. Nilsson, J. Salzer, A. Svenningsson, J. Mellergård, T. Olsson, E. Longinetti, T. Frisell, F. Piehl, Predictors of patient-reported fatigue symptom severity in a nationwide multiple sclerosis cohort, Mult Scler Relat Disord 70 (2023) 104481.

[69] J.J. Liu, X.S. Yuan, Z.M. Lu, Y. Liu, M.Y. Ni, W. Wang, L.H. Zhang, Z.N. Zhang, Z.Y. Wang, Y. Dong, The correlation between fatigue and depressive symptoms in patients with multiple sclerosis, Neurological Disease and Mental Health 23(5) (2023) 305-310. [Chinese]

**Supplementary Table 4** Characteristics of the included studies and their participants

| **First author,**  **publication year** | **Design** | **Survey year** | **Country** | **Mean Age(y)** | **Mean MS**  **Duration (y)** | **Mean EDSS score** | **Fatigue scale,**  **cut-off** | **Sample size, F/M (%)** | **Prevalence**  **Fatigue (%)** |
| --- | --- | --- | --- | --- | --- | --- | --- | --- | --- |
| Lobentanz 2004 | CC | 2003 | Austria | 50.6 | 15.8 | 5.8 | FSS>4 | 490, 72/28 | 80.0 |
| Tellez 2005 | CS | 2003 | Spain | 37.1 | 9.6 | 2♯ | FSS≥5 | 231, 69/31 | 55.0 |
| Hadjimichael 2008 | CS | 2002 | American | 47.6 | 12.8 | NR | FSS≥4 | 9077, 72/28 | 73.7 |
| Putzki 2008 | CS | 2004 | Germany | 43.5 | NR | NR | FSS≥5 | 320, 68/32 | 50.0 |
| Valko 2008 | CS | 2006 | Switzerland | 45 | 11.1 | 3.6 | FSS≥4 | 188, 67/33 | 69.1 |
| Koch 2009 | CS | 2008 | Holand | 49♯ | NR | 6♯ | FSS≥5 | 409, 70/30 | 69.9 |
| Morrow 2009 | CS | 2008 | American | 45.8 | NR | NR | FSS≥5 | 465, 77/23 | 57.2 |
| Glad 2011 | CS | 2003 | Norway | 53.9 | 22.2 | 4.7 | FSS≥4 | 188, 64/4 | 76.1 |
| Miletic 2011 | CS | 2007 | Serbia | 37.7 | 8.11 | 3.5 | FSS≥5 | 120, 65/35 | 67.5 |
| Dias 2012 | CS | 2011 | American | 45.8 | 11.7 | NR | FSS≥5 | 103, 72/28 | 53.4 |
| Kargarfard 2012 | CS | 2009 | Iran | 4.3 | 9.4 | 3.4 | MFIS≥45 | 281, 63/37 | 50.5 |
| Labuz 2012 | CS | 2010 | Poland | 37.7 | 6.7 | 2.2 | FSS>4 | 122, 71/29 | 61.5 |
| Chen 2013 | CS | 2011 | China | 39.1 | 12.3 | 2.3 | MFIS≥38 | 100, 78/22 | 52.0 |
| Wood 2013 | LC | 2004 | Australia | 48.2 | 12♯ | 3♯ | FSS≥5 | 192, 70/30 | 53.6 |
| Anens 2014 | CS | 2013 | Sweden | 51.5 | 11 ♯ | NR | FSS≥4 | 285, 71/29 | 62.1 |
| Clemenzi 2014 | CS | 2013 | Italy | 51.9 | 19.3 | 6.5 | FSS>4 | 133, 65/35 | 76.7 |
| Coyle 2014 | CS | 2013 | American | 49 | 9.5 | NR | FSS≥4 | 2966, 80/20 | 56.4 |
| Seyed 2014 | CS | 2010 | Iran | 32.4 | 6.3 | 2 | FSS≥4 | 160, 73/27 | 60.0 |
| Aygünoglu 2015 | CS | 2014 | Turkey | 34.2 | NR | 2.9 | FSS≥4 | 120, 70/30 | 70.0 |
| Braley 2015 | CS | 2013 | American | 47 | 10.2 | NR | FSS≥4 | 190, 67/33 | 63.7 |
| Kalron 2015 | CS | 2014 | Israel | 42.6 | 7.4 | 2.9 | MFIS≥38 | 124, 68/32 | 35.5 |
| Strober 2015 | CS | 2014 | American | 44.7 | 8.9 | NR | MFIS≥38 | 107, 86/14 | 46.7 |
| Weiland 2015 | CS | 2012 | American, Australia, UK, other | 45.5 | 8.5 | NR | FSS≥4 | 2118, 82/18 | 64.3 |
| Chahin 2015 | CS | 2014 | American | 48 | 10 | NR | MFIS≥38 | 139, 76/24 | 48.2 |
| Braga 2016 | CS | 2007 | Brazil | 44♯ | NR | NR | FSS≥28 | 122, 66/34 | 68.9 |
| Leonavicius 2016 | CS | 2014 | Lithuania | 44.7 | 12.6 | 3.8 | FSS≥4 | 137, 72/28 | 68.6 |
| Ma 2017 | CS | 2015 | China | 40.2 | 4.9 | NR | FSS≥4 | 231, 58/42 | 48.5 |
| Marck 2017 | CS | 2012 | Australia | 45.5 | NR | NR | FSS≥4 | 2128, 82/18 | 65.6 |
| Nociti 2017 | CS | 2014 | Italy | 43.1 | 10.6 | 2.4 | MFIS≥38 | 102, 64/4 | 28.4 |
| Reilly 2017 | CS | 2012 | Australia,  New Zealand, American, Canada | 45.7 | NR | NR | FSS≥4 | 2079, 83/17 | 65.6 |
| Rosti 2017 | CS | 2016 | Finland | 53.8 | 16.4 | 4 | FSS≥4 | 553, 79/21 | 65.1 |
| Heffer 2018 | CS | 2017 | UK | 43.3 | 7.3 | NR | FSS≥4 | 132, 84/16 | 85.6 |
| van Gorp 2018 | CS | 2017 | Holand | 42.5♯ | NR | NR | MFIS≥38 | 163, 77/23 | 42.3 |
| Carnero 2019 | CS | 2018 | Argentina | 39.4 | 7.5 | 2.6 | FSS≥5 | 202, 68/32 | 52.0 |
| Phyo 2019 | CS | 2012 | New Zealand | 48.3 | NR | NR | FSS≥4 | 191, 79/21 | 63.4 |
| Rooney 2019 | CS | 2018 | UK, American, Australia, other | 46 | 9.6 | NR | FSS≥5 | 412, 81/19 | 68.7 |
| Schmidt 2019 | CS | 2012 | Germany | 44.5 | 10.6 | 3.3 | FSMC≥43 | 260, 80/20 | 68.1 |
| Silva 2019 | CS | 2015 | Brazil | 39.9 | NR | NR | MFIS≥38 | 100, 78/22 | 63.0 |
| Alshamrani 2020 | CS | 2019 | Saudi Arabia | NR | NR | NR | MFIS≥38 | 465, 63/37 | 84.9 |
| Contentti 2020 | CS | 2019 | Argentina | 39.9 | NR | NR | FSS≥5 | 301, 71/29 | 43.5 |
| Eizaguirre 2020 | CS | 2019 | Argentina | 40 | 10.1 | 2.4 | FSS≥4 | 128, 59/41 | 51.6 |
| Neal 2020 | CS | 2017 | American | 59.3 | 20.3) | NR | FSS≥4 | 252, 81/19 | 69.0 |
| Purmonen 2020 | CS | 2015 | Finland | 53.8 | NR | NR | FSS≥4 | 498, 78/22 | 65.7 |
| Rzepka 2020 | CS | 2017 | Poland | 38.8 | 8.2 | 2.5 | FSS≥4 | 100, 78/22 | 42.0 |
| Wallis 2020 | CS | 2012 | Holand | 47.8 | 7.4 | 3.5 | FSS≥4 | 119, 63/37 | 88.2 |
| Bakirtzis 2020 | CC | 2019 | Greece | 4.7 | NR | NR | MFIS≥38 | 120, 63/37 | 37.5 |
| Marchesi 2020 | CC | 2018 | Italy | 43.1 | 13♯ | NR | MFIS≥38 | 46, 62/38 | 47.5 |
| Broch 2021 | CS | 2018 | Norway | 52.1 | 10♯ | 2.5 ♯ | FSMC≥43 | 1454, 70/30 | 81.3 |
| Carnero1 2021 | CS | 2017 | Argentina | 38.4 | NR | NR | FSS≥5 | 167, 71/29 | 49.7 |
| Carnero2 2021 | CS | 2019 | Argentina | 38.6 | 7.3 | 2 | FSS≥5 | 249, 75/25 | 40.6 |
| Gustavsen 2021 | CS | 2018 | Denmark | 49.4 | 11.7 | NR | FSS≥4 | 2009, 69/31 | 63.8 |
| Kamudoni 2021 | CS | 2019 | UK | 49.9 | 10.2 | NR | FSS≥4 | 374, 76/24 | 76.2 |
| Moore 2022 | CS | 2017 | UK | 54.7 | 13.2 | NR | FSS≥5 | 775, 74/26 | 55.1 |
| Abdulla 2022 | CS | 2018 | Saudi Arabia | 34.7 | 4.1 | 3.3 | MFIS> 35.5 | 175, 78/22 | 56.6 |
| AlSaeed 2022 | CS | 2021 | Saudi Arabia | NR | 5.5 | NR | FSS≥5 | 323, 70/30 | 48.3 |
| Bellew 2022 | CS | 2020 | Ireland | 42.9 | 9.5 | NR | MFIS≥38 | 192, 69/31 | 57.8 |
| Davenport 2022 | CS | 2020 | Ireland | 42.5 | 9.2 | NR | MFIS≥38 | 177, 70/30 | 46.9 |
| Ozdogar 2022 | CS | 2021 | Israel, Turkey | 40 | 6 | 2 | MFIS≥38 | 990, 65/35 | 39.8 |
| Picariello 2022 | CS | 2021 | UK | 55.1 | 19.2 | 5 | FSS≥5 | 4238, 74/26 | 63.0 |
| Royer 2022 | CS | 2020 | France | 49.5 | NR | NR | MFIS≥38, FSS≥4 | 191, 70/30 | 57.6 |
| Saul 2022 | CS | 2021 | Australia | 44.5 | NR | NR | FSS≥5 | 190, 81/19 | 45.3 |
| Valentine 2022 | CS | 2016 | American, Sweden | 39 | NR | NR | FSS≥4 | 230, 71/29 | 73.5 |
| Abou 2023 | CS | 2023 | American | 45 | 11♯ | NR | FSS≥4 | 537, 79/21 | 34.6 |
| Ahvenjärvi 2023 | CS | 2018 | Finland | 45 | NR | NR | FSMC≥43 | 198, 82/18 | 73.2 |
| Biasi 2023 | CS | 2021 | Italy | 40.4 | 10.2 | NR | FSS≥4.5 | 200, 64/4 | 71.0 |
| Donzé 2023 | CS | 2015 | France | 48.1 | 15.3 | NR | EMIF-SEP ≥55 | 352, 70/30 | 42.3 |
| Englund 2023 | CS | 2015 | Sweden | 41.6 | 10.6 | 1.9 | FSMC≥43 | 3213, 70/30 | 59.3 |
| Liu 2023 | CS | 2020 | China | 43.9 | NR | NR | MFIS≥38 | 108, 55/45 | 50.9 |
| Bateman 2023 | CC | 2021 | Australia | 47.8 | 9.8 | 3.2 | FSS≥5 | 103, 77/23 | 46.6 |

Note. Studies are ordered by publication date.

Abbreviation: CS, cross-sectional study; EDSS, Expanded Disability Status Scale; EMIF-SEP, French version of the Fatigue Impact Scale; F, Female; FSMC, Fatigue Scale for Motor and Cognitive Functions; FSS, Fatigue Severity Scale; LS, longitudinal study; M, Male; MFIS, Modified Fatigue Impact Scale; MS, Multiple sclerosis; NR, not reported; SD, standard deviation; UK, United Kingdom.

♯Median(IQR)

**Supplementary Table 5** Risk of bias assessment of studies included based on the AHRQ tool

| **Author, year** | **Item 1** | **Item 2** | **Item 3** | **Item 4** | **Item 5** | **Item 6** | **Item 7** | **Item 8** | **Item 9** | **Item 10** | **Item 11** | **Total score** | **ROB** |
| --- | --- | --- | --- | --- | --- | --- | --- | --- | --- | --- | --- | --- | --- |
| Tellez 2005 | 1 | 0 | 1 | 0 | 0 | 1 | 1 | 1 | 0 | 1 | 1 | 7 | Moderate |
| Hadjimichael 2008 | 1 | 0 | 1 | 1 | 0 | 0 | 0 | 1 | 0 | 1 | 1 | 6 | Moderate |
| Putzki 2008 | 1 | 1 | 1 | 1 | 0 | 0 | 1 | 1 | 0 | 1 | 1 | 8 | Low |
| Vaklo 2008 | 1 | 0 | 1 | 0 | 0 | 0 | 1 | 1 | 1 | 1 | 1 | 7 | Moderate |
| Koch 2009 | 1 | 0 | 0 | 0 | 0 | 0 | 1 | 1 | 0 | 1 | 1 | 5 | Moderate |
| Morrow 2009 | 1 | 0 | 0 | 0 | 1 | 0 | 1 | 1 | 1 | 0 | 1 | 6 | Moderate |
| Glad 2011 | 1 | 1 | 1 | 1 | 1 | 0 | 1 | 1 | 0 | 0 | 1 | 8 | Low |
| Miletic 2011 | 1 | 1 | 1 | 0 | 1 | 0 | 1 | 1 | 0 | 1 | 1 | 8 | Low |
| Dias 2012 | 1 | 1 | 1 | 1 | 1 | 0 | 1 | 1 | 0 | 1 | 1 | 9 | Low |
| Kargarfard 2012 | 1 | 0 | 1 | 1 | 1 | 0 | 1 | 1 | 0 | 1 | 1 | 8 | Low |
| Labuz-Roszak 2012 | 1 | 0 | 1 | 0 | 1 | 0 | 1 | 0 | 1 | 1 | 1 | 7 | Moderate |
| Chen 2013 | 1 | 1 | 1 | 0 | 1 | 0 | 1 | 0 | 1 | 0 | 1 | 7 | Moderate |
| Anens 2014 | 1 | 1 | 0 | 1 | 0 | 0 | 1 | 0 | 1 | 1 | 1 | 7 | Moderate |
| Clemenzi 2014 | 1 | 1 | 1 | 1 | 1 | 1 | 1 | 0 | 0 | 1 | 1 | 9 | Low |
| Coyle 2014 | 1 | 1 | 0 | 1 | 0 | 1 | 1 | 1 | 0 | 1 | 0 | 7 | Moderate |
| Seyed 2014 | 1 | 1 | 1 | 1 | 0 | 0 | 1 | 1 | 1 | 1 | 1 | 9 | Low |
| Aygünoglu 2015 | 1 | 1 | 0 | 1 | 1 | 0 | 1 | 0 | 1 | 1 | 1 | 8 | Low |
| Braley 2015 | 1 | 1 | 1 | 0 | 1 | 0 | 1 | 1 | 0 | 1 | 1 | 8 | Low |
| Kalron 2015 | 1 | 1 | 0 | 1 | 0 | 1 | 0 | 1 | 1 | 0 | 1 | 7 | Moderate |
| Strober 2015 | 1 | 1 | 0 | 1 | 0 | 0 | 0 | 1 | 1 | 0 | 1 | 6 | Moderate |
| Weiland 2015 | 1 | 1 | 1 | 1 | 0 | 0 | 0 | 1 | 1 | 1 | 1 | 8 | Low |
| Braga 2016 | 1 | 1 | 1 | 0 | 1 | 0 | 0 | 0 | 1 | 1 | 1 | 7 | Moderate |
| Leonavicius 2016 | 1 | 1 | 1 | 0 | 1 | 0 | 1 | 1 | 0 | 1 | 1 | 8 | Low |
| Ma 2017 | 1 | 1 | 1 | 1 | 0 | 0 | 1 | 1 | 0 | 1 | 1 | 8 | Low |
| Marck 2017 | 1 | 1 | 1 | 1 | 0 | 0 | 0 | 1 | 1 | 0 | 1 | 7 | Moderate |
| Nociti 2017 | 1 | 1 | 1 | 1 | 1 | 0 | 0 | 1 | 1 | 1 | 1 | 9 | Low |
| Reilly 2017 | 1 | 1 | 1 | 1 | 0 | 0 | 0 | 1 | 0 | 0 | 1 | 6 | Moderate |
| Rosti 2017 | 1 | 1 | 0 | 1 | 0 | 1 | 0 | 1 | 1 | 1 | 1 | 8 | Low |
| Heffer 2018 | 1 | 1 | 0 | 1 | 0 | 1 | 0 | 0 | 0 | 0 | 1 | 5 | Moderate |
| van-Gorp 2018 | 1 | 1 | 0 | 0 | 1 | 0 | 1 | 1 | 0 | 1 | 1 | 7 | Moderate |
| Carnero 2019 | 1 | 1 | 1 | 1 | 0 | 1 | 0 | 1 | 1 | 0 | 1 | 8 | Low |
| Phyo 2019 | 1 | 1 | 1 | 1 | 0 | 0 | 0 | 1 | 0 | 1 | 1 | 7 | Moderate |
| Rooney 2019 | 1 | 1 | 1 | 1 | 0 | 0 | 0 | 1 | 1 | 1 | 1 | 8 | Low |
| Schmidt 2019 | 1 | 1 | 1 | 0 | 1 | 1 | 1 | 1 | 1 | 1 | 1 | 10 | Low |
| Silva 2019 | 1 | 1 | 1 | 0 | 1 | 0 | 0 | 1 | 1 | 0 | 1 | 7 | Moderate |
| Alshamrani 2020 | 1 | 1 | 1 | 1 | 1 | 0 | 1 | 0 | 0 | 1 | 1 | 8 | Low |
| Contentti 2020 | 1 | 1 | 1 | 1 | 0 | 0 | 1 | 1 | 1 | 1 | 1 | 9 | Low |
| Eizaguirre 2020 | 1 | 1 | 0 | 0 | 1 | 0 | 0 | 1 | 1 | 0 | 1 | 6 | Moderate |
| Neal 2020 | 1 | 1 | 1 | 1 | 0 | 0 | 1 | 1 | 1 | 1 | 1 | 9 | Low |
| Purmonen 2020 | 1 | 1 | 1 | 1 | 0 | 0 | 1 | 1 | 0 | 0 | 1 | 7 | Moderate |
| Rzepka 2020 | 1 | 1 | 1 | 0 | 1 | 0 | 1 | 0 | 1 | 0 | 1 | 7 | Moderate |
| Wallis 2020 | 1 | 1 | 1 | 0 | 1 | 0 | 1 | 1 | 0 | 1 | 1 | 8 | Low |
| Broch 2021 | 1 | 1 | 1 | 1 | 0 | 0 | 1 | 1 | 1 | 1 | 1 | 9 | Low |
| Carnero 2021 | 1 | 1 | 1 | 1 | 0 | 0 | 1 | 1 | 0 | 1 | 1 | 8 | Low |
| Carnero-2 2021 | 1 | 1 | 1 | 1 | 0 | 0 | 1 | 1 | 0 | 1 | 1 | 8 | Low |
| Gustavsen 2021 | 1 | 0 | 1 | 1 | 0 | 0 | 1 | 1 | 0 | 1 | 1 | 7 | Moderate |
| Kamudoni 2021 | 1 | 1 | 1 | 1 | 0 | 0 | 0 | 1 | 0 | 0 | 1 | 6 | Moderate |
| Moore 2022 | 1 | 1 | 1 | 1 | 0 | 0 | 1 | 1 | 1 | 1 | 0 | 8 | Low |
| Abdulla 2022 | 1 | 1 | 1 | 0 | 1 | 0 | 1 | 1 | 0 | 1 | 1 | 8 | Low |
| AlSaeed 2022 | 1 | 1 | 0 | 1 | 0 | 0 | 1 | 0 | 1 | 1 | 1 | 7 | Moderate |
| Bellew 2022 | 1 | 1 | 1 | 0 | 1 | 0 | 1 | 1 | 1 | 0 | 1 | 8 | Low |
| Davenport 2022 | 1 | 1 | 1 | 0 | 1 | 1 | 0 | 1 | 0 | 0 | 1 | 7 | Moderate |
| Ozdogar 2022 | 1 | 1 | 0 | 1 | 1 | 0 | 0 | 1 | 1 | 0 | 1 | 7 | Moderate |
| Picariello 2022 | 1 | 1 | 1 | 1 | 0 | 0 | 1 | 1 | 1 | 1 | 1 | 9 | Low |
| Royer 2022 | 1 | 1 | 1 | 1 | 0 | 0 | 1 | 1 | 1 | 1 | 1 | 9 | Low |
| Saul 2022 | 1 | 1 | 1 | 1 | 1 | 0 | 1 | 1 | 0 | 1 | 1 | 9 | Low |
| Valentine 2022 | 1 | 1 | 1 | 1 | 1 | 0 | 1 | 0 | 0 | 1 | 1 | 8 | Low |
| Abou 2023 | 1 | 1 | 1 | 1 | 0 | 0 | 0 | 1 | 1 | 1 | 1 | 8 | Low |
| Ahvenjärvi 2023 | 1 | 1 | 1 | 0 | 1 | 0 | 0 | 0 | 1 | 1 | 1 | 7 | Moderate |
| Biasi 2023 | 1 | 1 | 1 | 0 | 1 | 0 | 1 | 1 | 1 | 0 | 1 | 8 | Low |
| Donzé 2023 | 1 | 1 | 1 | 1 | 1 | 0 | 1 | 1 | 0 | 1 | 1 | 9 | Low |
| Englund 2023 | 1 | 1 | 1 | 1 | 1 | 0 | 1 | 1 | 1 | 1 | 1 | 10 | Low |
| Liu 2023 | 1 | 1 | 1 | 0 | 1 | 0 | 1 | 1 | 1 | 0 | 1 | 8 | Low |
| Chahin 2015 | 1 | 1 | 0 | 0 | 1 | 0 | 0 | 1 | 0 | 0 | 1 | 5 | Moderate |

**Note.** ROB, Risk of bias

Scored as 1: for “yes” and 0 for “no” or “unclear”

**Item1**: Whether the data source is clear

**Item2**: Whether the inclusion and exclusion criteria of the exposed and non-exposed groups (cases and controls) are clear or refer to previous **public**ations

**Item3**: Whether the collection time of the study subjects is clear

**Item4**: Whether the continuity of the study subjects is clear

**Item5**: Whether other aspects of the study subjects are affected by the subjective factors of the evaluators

**Item6**: Whether any evaluation measures for quality assurance described

**Item7**: Whether the reasons for excluding the subjects are described

**Item8**: Whether the measures to evaluate/control the confounding factors are evaluated

**Item9**: Whether the treatment of lost data is described

**Item10**: Summarize patient response rates and completeness of data collection

**Item11**: If there is follow-up, is whether the expected percentage of patients with incomplete data or follow-up results are describe

**Supplementary Table 6** Risk of bias assessment of studies included based on the NOS tool

| **Author, year** | **Selection** | | | | **Comparability** | **Outcome** | | | **Total score** | **ROB** |
| --- | --- | --- | --- | --- | --- | --- | --- | --- | --- | --- |
|  | **Item 1** | **Item 2** | **Item 3** | **Item 4** | **Item 5** | **Item 6** | **Item 7** | **Item 8** |  |  |
| Wood 2013 | 1 | 1 | 1 | 0 | 1 | 0 | 1 | 1 | 6 | Moderate |
| Schließeit 2021 | 1 | 0 | 1 | 1 | 1 | 0 | 1 | 1 | 6 | Moderate |
| Lobentanz 2004 | 1 | 1 | 1 | 1 | 1 | 0 | 0 | 0 | 5 | Moderate |
| Bateman 2023 | 0 | 0 | 0 | 1 | 2 | 1 | 1 | 0 | 5 | Moderate |
| Bakirtzis 2020 | 1 | 1 | 0 | 1 | 1 | 1 | 1 | 0 | 6 | Moderate |

**Note**. ROB, Risk of bias

Each study was given a maximum of 1 point for each entry on “selection” and “outcomes” and a maximum of 2 points for “comparability”.

**Item1**: representative of the exposed cohort

**Item2**: selection of the non-exposed cohort

**Item3**: ascertainment of exposure

**Item4**: demonstration that outcome of interest was not present at start of study

**Item5**: comparability of cohorts on the basis of the design or analysis Item6: assessment of outcome

**Item7**: was follow-up long enough for outcomes to occur

**Item8**: adequacy follow-up cohorts

**Supplementary Table 7** Prevalence of fatigue and by sex, phenotypes, and education

| **Category** | **No. of studies** | **Total  sample size** | **Prevalence**  **(95 % CI) (%)** | **Heterogeneity *I*^2^(%)** | ***P*-value** |
| --- | --- | --- | --- | --- | --- |
| Overall | 69 | 44468 | 59.1(55.9-62.2) | 97.3 | < 0.001 |
| Gender |  |  |  |  |  |
| Female | 15 | 6982 | 58(51.4-64.5) | 96.6 | < 0.001 |
| Male | 15 | 2598 | 56.5(49.6-63.4) | 91.4 | < 0.001 |
| MS type |  |  |  |  |  |
| RRMS | 15 | 10897 | 54.7(48.5-60.9) | 96.7 | < 0.001 |
| SPMS | 10 | 5734 | 74.4(64.3-83.3) | 94.9 | < 0.001 |
| PPMS | 8 | 1253 | 64.3(55.1-73.5) | 86.5 | < 0.001 |
| Education |  |  |  |  |  |
| ≤ 12years | 4 | 2109 | 64.3(52-76.6) | 89.7 | < 0.001 |
| > 12years | 4 | 3309 | 47.9(36-59.9) | 93.1 | < 0.001 |

Note. CI, confidence interval; PPMS, primary progressive MS; RRMS, Relapsing-remitting MS; SPMS, Secondary progressive MS.
